# Isolation and Characterization of Live Yeast Cells from Ancient Vessels as a Tool in Bio-Archaeology

**DOI:** 10.1128/mBio.00388-19

**Published:** 2019-04-30

**Authors:** Tzemach Aouizerat, Itai Gutman, Yitzhak Paz, Aren M. Maeir, Yuval Gadot, Daniel Gelman, Amir Szitenberg, Elyashiv Drori, Ania Pinkus, Miriam Schoemann, Rachel Kaplan, Tziona Ben-Gedalya, Shunit Coppenhagen-Glazer, Eli Reich, Amijai Saragovi, Oded Lipschits, Michael Klutstein, Ronen Hazan

**Affiliations:** a Institute of Dental Sciences, Faculty of Dental Medicine, Hebrew University of Jerusalem, Jerusalem, Israel; b Israel Antiquities Authority, Jerusalem, Israel; c Tell es-Safi/Gath Archaeological Project, The Martin (Szusz) Department of Land of Israel and Archaeology, Bar-Ilan University, Ramat-Gan, Israel; d Tel Aviv University, Tel Aviv, Israel; e Microbial Metagenomics Division, Dead Sea and Arava Science Center, Masada, Israel; f Department of Chemical Engineering and Biotechnology, Ariel University and Eastern R&D Center, Ariel, Israel; g Department of Molecular Biology, Ariel University and Eastern R&D Center, Ariel, Israel; h Lautenberg Center for Immunology and Cancer Research, Faculty of Medicine, Hebrew University of Jerusalem, Jerusalem, Israel; Hebrew University of Jerusalem

**Keywords:** ancient fermented food and beverages, ancient pottery vessels, beer, bio-archaeology, experimental archaeology, yeasts

## Abstract

Ancient fermented food has been studied based on recipes, residue analysis, and ancient-DNA techniques and reconstructed using modern domesticated yeast. Here, we present a novel approach based on our hypothesis that enriched yeast populations in fermented beverages could have become the dominant species in storage vessels and their descendants could be isolated and studied today. We developed a pipeline of yeast isolation from clay vessels and screened for yeast cells in beverage-related and non-beverage-related ancient vessels and sediments from several archaeological sites. We found that yeast cells could be successfully isolated specifically from clay containers of fermented beverages. The findings that genotypically the isolated yeasts are similar to those found in traditional African beverages and phenotypically they grow similar to modern beer-producing yeast strongly suggest that they are descendants of the original fermenting yeast. These results demonstrate that modern microorganisms can serve as a new tool in bio-archaeology research.

## Abstract (precis)

So far, most of the study of ancient organisms has been based mainly on the analysis of ancient DNA. Here we show that it is possible to isolate and study microorganisms—yeast in this case—from ancient pottery vessels used for fermentation. We demonstrate that it is highly likely that these cells are descendants of the original yeast strains that participated in the fermentation process and were absorbed into the clay matrix of the pottery vessels. Moreover, we characterized the isolated yeast strains, their genomes, and the beer they produced. These results open new and exciting avenues in the study of domesticated microorganisms and contribute significantly to the fields of bio- and experimental archaeology that aim to reconstruct ancient artifacts and products.

## INTRODUCTION

Experimental archaeology is a field of research that studies ancient cultures by trying to reconstruct ancient lifestyles, including tools, housing, clothing, and diet (1, 2). Among the most challenging subjects of study in this field are fermented food products, such as cheese and pickles, and alcoholic beverages, including wine, beer, and mead (honey wine). All of these products played important roles in ancient societies (3), as central components of ancient diets, which is especially important due to their preservation under diverse conditions. In particular, alcoholic beverages fulfilled various important social, political, economic, and religious functions (4). In fact, alcohol has served throughout history, and continues today, as an important “social lubricant” in diverse human social and political contexts (5–9). There is sundry archaeological evidence of fermented beverages, as well as their production and consumption, in ancient societies throughout the world, from late Prehistoric periods onward (10). Extensive evidence of wine and beer production in Egypt, Mesopotamia, and the Near East as early as the mid-4th millennium BCE (Before Common Era) has been discovered (4, 11, 12). This includes textual evidence in the form of administrative lists and narratives that mention such beverages, including actual recipes of different types of wine (13) and beer, as well as small-scale models and paintings of their production (4, 14). Similarly, chemical evidence of wine and beer production has been found in various components of breweries (15), including vessels and related installations. Such residue analyses have enabled the identification of alcoholic beverages of numerous cultures as early as the Neolithic period (ca. 6000 to 5000 BC) in the region of modern Georgia (16), and ancient China (17, 18), Mediterranean France (19), Cyprus (14), Bronze and Iron Age Israel (13), Nordic cultures of Scandinavia (20), early Celts in Germany (21), early cultures of the Andes (8), Prehistoric Europe and Indo-Iranian Asia (22), and ancient Egypt (23), leading in some cases to the identification of specific compositions of these beverages (9, 10, 24).

Based on this evidence, there have been several attempts to recreate ancient beer and wine, but those were always brewed using modern ingredients combined with modern domesticated commercial yeast (predominantly Saccharomyces cerevisiae) (4, 21) and not with the actual microorganisms that might have been used in the production of these fermented beverages. On the other hand, up until now, the study of ancient microorganisms, including bacteria (25, 26), viruses (27), and yeast (28), has mainly focused on ancient DNA studies.

Here, we isolated yeast directly from ancient vessels that had previously been suggested to have served as beverage containers. We found that yeast are significantly more abundant in these putative beverage containers than in other non-beverage-related archaeological vessels, from these and other sites, or in sediments from these sites and the surrounding environment. This supports the hypothesis that the yeast found in the beverage containers originated from the large amount of yeast cells that grew during the beverage fermentation, continued to reproduce, and survived as colonies in the microenvironments of the pores in the ceramic matrix of these vessels. In agreement with this hypothesis, phenotypic and genomic characterization of these yeast strains, including genomic DNA sequencing, showed that they are similar to yeast found in modern traditional beers and are able to ferment and produce drinkable beer similar to modern beverages.

## RESULTS

### Isolation of yeast strains from ancient vessels.

We hypothesized that the enrichment of clay vessels with large amounts of fermenting yeast that were absorbed into the vessel pores of the ceramic matrix permanently changed the vessel's microorganism content (vessel microbiome).

**FIG 1:**
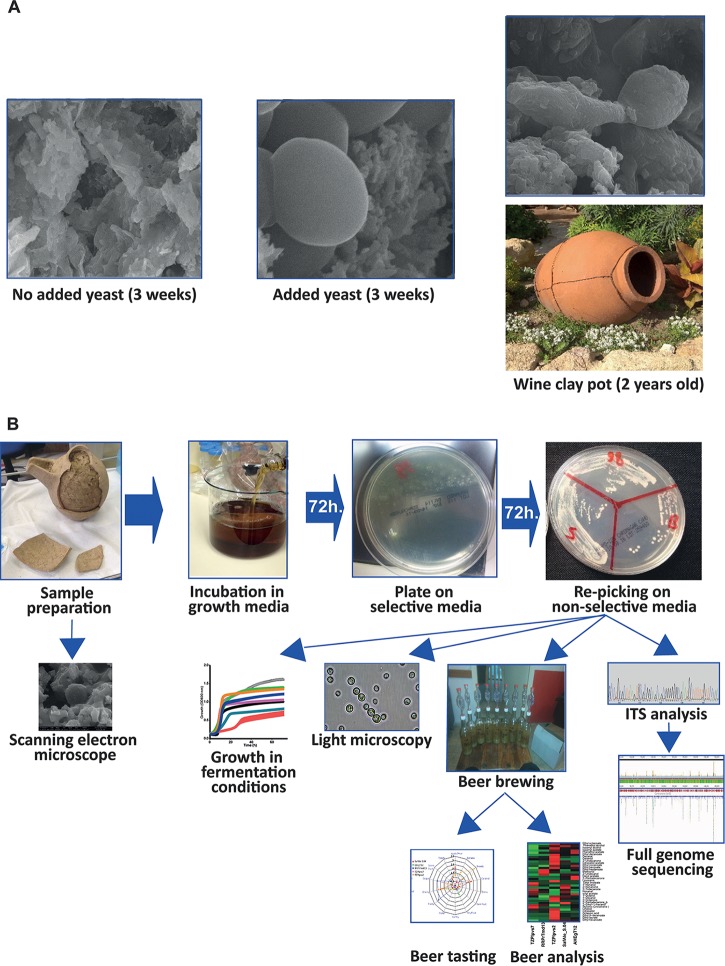
Isolation of yeast from clay vessels. (A) Yeast strains in clay vessels. Scanning electron microscope (SEM) pictures of the inside of a modern clay vessel buried in the ground for 3 weeks without beer (left panel) and presoaked with unfiltered beer (middle panel) prior to burial. On the right panel is a 2-year out-of-use wine clay vessel (bottom) that yielded live yeast cells, observed as colonies and by electron microscopy (EM [upper panel]). Yeast cells were only successfully isolated from the last two vessels. (B) The pipeline of yeast isolation and characterization from vessels. Putative fermented beverage-containing vessels were carefully dismantled. Small pieces were sent for scanning electron microscopy (SEM), and the rest were incubated in growth medium (YPD) for 72 h at room temperature. Samples were plated on selective plates with antibiotics to eliminate bacteria. After 72 h, yeast colonies appeared and were regrown on new plates. The yeast strains were taken for various analyses, including full-genome sequencing and comparison of growth under fermentation-related conditions in beer wort. In addition, beer was brewed according to a standard recipe using the isolated yeast strains. The presence of aromatic and flavor compounds in the beers was analyzed quantitatively, and their flavor was qualitatively evaluated by specialized beer tasters.

Indeed, testing of several modern vessels that were filled with filtered and unfiltered beer and buried for 3 weeks underground, as well as further tests of a clay wine vessel that had not been used for more than 2 years, revealed that yeast cells can be found in the clay matrix, after an extended period of time (Fig. 1A). Next, we tested several methods of yeast isolation and developed a pipeline (Fig. 1B) that enabled us to efficiently isolate viable yeast cells from these modern clay containers. In contrast, we could not isolate any live yeast from the control vessels, which were filled with filtered beer, nor were yeast cells detected by electron microscopy (Fig. 1A, left panel).

**FIG 2:**
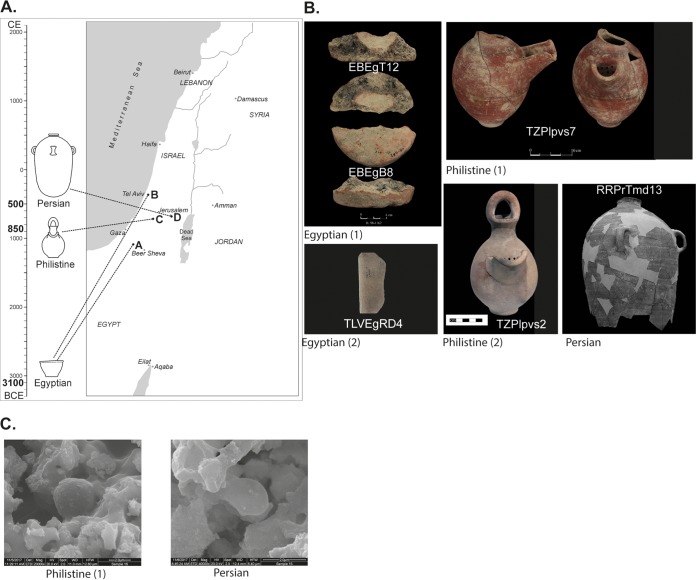
Ancient vessels that putatively contained fermented beverage and were used for yeast isolation. (A) A map and timeline of the archaeological sites from which the vessels yielding fermenting yeast strains were excavated. (B) Photographs of the vessels. The white text indicates the name of the yeast strain isolated, and the text below the photographs denotes the archaeological culture with which the vessels are associated. (C) Representative SEM image of vessels with “yeast-like” structures (compared to Fig. 1A).

Next, we tested ancient ceramic vessels from three different historical periods, found in four different archaeological sites located in Israel (Fig. 2A). Each of these sites contained vessels that were assumed to have been associated with fermented beverages (Fig. 2B), based on ancient iconography, functional analysis based on the vessels’ shape, or previously conducted organic residue analysis. Electron microscopy visualization showed “yeast-like” structures (Fig. 2C) similar to those of the modern clay vessels (Fig. 1A), which prompted us to try isolating live yeast cells from the ancient vessels.

The first vessels were excavated from two sites dated to the Early Bronze Age IB (ca. 3100 BCE). The first site is En-Besor in the northwestern Negev desert, a site relating to the Egyptian activities in southern Canaan during the late 4th millennium BCE, as evidenced by typical Egyptian architecture, pottery, and clay bullae with hieroglyphic symbols (29, 30). The second site was recently excavated at Ha-Masger Street in Tel Aviv and contained basin fragments, typical of Egyptian-style breweries, perhaps evidence of an Egyptian enclave within a local Canaanite settlement. We tested five ceramic fragments (Fig. 2A and B) of vessels from these two sites, which according to ancient Egyptian depictions were used as beer basins (4). These vessel fragments yielded three yeast strains, two from En-Besor, and one from Ha-Masger St., designated EBEgT12, EBEgB8, and TLVEgRD4 (Table 1).

**TABLE 1:** Genetic identification of yeast strains isolated from ancient vessels^*a*^

Isolate (common name)^*b*^	NCBI accession no.	Site	Period	Vessel type	Culture	Closest relative	Status	LSU rRNA^*c*^		Phylogenomic tree^*d*^		Whole-genome BLAST^*e*^		Relation to fermented beverages	Reference(s)
								Tree distance	*P* value (known sp.)	Position	Node support (BP)^*f*^	Match	Scaffold prop^*g*^		
EBEgT12 (T12)	SAMN08918525	Ein-Besor, North Negev	EB IB	Putative fermented liquid container	Egyptian	*N. delphensis*^*h*^	sp. nov.	0.023	0.02	Very similar, sister node of *N. delphensis*	100	*N. delphensis*	>0.9	Isolated from dry figs and identified in screen for ethanol- fermenting yeast	40, 88, 89
EBEgB8 (B8)	SAMN08915826	Ein-Besor, North Negev	EB IB	Putative fermented liquid container	Egyptian	*N. delphensis*^*h*^		NA	NA	Very similar, sister node of *N. delphensis*		*N. delphensis*	0.5	Isolated from dry figs and identified in screen for ethanol- fermenting yeast	40, 88, 89
TLVEgRD4 (Red4)	SAMN08918530	Hamasger St., Tel Aviv	EB IB	Putative fermented liquid container	Egyptian	*R. glutinis*	Conspecific	2 × 10^−6^	1	NA	NA	NA	NA	Isolated from locally fermented beverages sold in Nigeria; known as a beer contaminant	43
TZPlpvs7 (PVS7)	SAMN08918531	Tell es-Safi/ Gath	Iron IIA	Putative beer jug	Philistine	*Debaryomycetaceae* sp.	sp. nov., putative	NA	NA	Nested within *Debaryomycetaceae*, very distantly related to *Priceomyces*	100	*Debaryomycetaceae*	0.9	Found in African traditional beers brewed with sorghum malt	44, 45
TZPlpvs2 (PVS2)	SAMN08918658	Tell es-Safi/ Gath	Iron IIA	Putative beer jug	Philistine	S. cerevisiae	Conspecific	NA	NA	Within S. cerevisiae	100	S. cerevisiae	0.6	Main brewing yeast	41
RRPrTmd13 (Temed)	SAMN08918675	Ramat Rachel, Jerusalem	Persian	Mead container	Persian	*H. burtonii*^*i*^	sp. nov., putative	NA	NA	Very similar, sister node of *H. burtonii*	100	*H. burtonii*	>0.95	Isolated from tej, an Ethiopian honey wine	22, 46

a Shown is a summary of the phylogenetic identification of the yeast strains isolated from ancient putative beverage vessels and the source, site, and period of the vessels.

b Strains’ systematic nomenclature. The first letters represent the archaeological site: EB, Ein-Besor; TLV, Tel Aviv; Tz, Tell es-Safi (known also as Tel Zafit); and RR, Ramat Rachel. The next two letters denote the culture that produced the vessel: Eg, Egyptian; Pl, Philistine; and Pr, Persian. The last letters and number keep the common short name originally given to the yeast strain in the lab, mentioned also in parentheses after the full names.

c “LSU rRNA” represents the patristic distance (substitutions per base) between the isolate and its closest relative, given with the probability that this is an intraspecific tree distance.

d Shown is the phylogenetic position of the isolate in the phylogenomic tree, provided with the branch support of this relationship. Node supports are bootstrap percentages.

e Shown are the closest BLAST match of most contigs and the proportion of contigs that are assigned to this match. See the supplemental material for description of percentages of identities of the closest matches.

f “Node support (BP)” represents the percentage of the bootstrap tree that had the same topology as the maximum likelihood tree for a given node.

g “Scaffold prop” represents the proportion of scaffolds that had the taxon as their first BLAST hit.

h Nakaseomyces delphensis has the following synonyms: *Saccharomyces delphensis*, Dekkeromyces delphensis, Guilliermondella delphensis, Kluyveromyces delphensis, and Zygofabospora delphensis (40, 90; http://www.mycobank.org/name/Nakaseomyces%20delphensis).

i *Hyphopichia burtonii* has the following synonyms*:* Pichia burtonii, Endomycopsis burtonii Boidin, Candida armeniaca-cornusmas, Candida fibrae Nakase, Cladosporium fermentans, Sporotrichum anglicum, Sporotrichum carougeaui, Trichosporon behrendii, and Trichosporon beijingense (http://www.mycobank.org/Biolomics.aspx?Table=Mycobank&Rec=36231&Fields=All).

The third site sampled was Philistine Tell es-Safi/Gath (in central Israel), specifically from contexts dating to the Iron IIA (ca. 850 BCE) (31, 32). The Philistines, one of the so-called “Sea Peoples,” were an important culture in the Levant during the Iron Age (ca. 1200 to 600) and are often mentioned in the Bible as enemies of the Israelites (33). At the time, Philistine Gath was the largest and most important Philistine site in the region (31). We tested 12 samples from two well-preserved Philistine jugs (Fig. 2B; see Table S1 in the supplemental material) of a type usually associated with beer or other fermented alcoholic drinks, based on their spout and a strainer spout on their side (34–36). Each of these vessels yielded a yeast strain, designated TZPlpvs7 and TZPlpvs2 (Table 1).

10.1128/mBio.00388-19.5 TABLE S1 List of samples tested for the presence of yeast. Listed are samples from sites in Israel tested for the presence of live yeast cells. (A) Samples from ancient vessels thought to contain alcoholic beverages based upon their structure, archaeological context, or residue analysis excavated in En-Besor, Ha-Masger St., Tell es-Safi/Gath, and Ramat Rachel. (B) Control samples of beverage-unrelated vessels and sediments and stones from the archaeological sites and from agricultural land near the sites (Ma'on). (C) Oil lamps that initially served as controls, but surprisingly yielded a significant amount of live yeast cells, most probably originating from the olives used for the oil making. Download Table S1, DOCX file, 0.1 MB. Copyright © 2019 Aouizerat et al. 2019 Aouizerat et al. This content is distributed under the terms of the Creative Commons Attribution 4.0 International license.

The fourth site was Ramat Rachel, located between Jerusalem and Bethlehem (Fig. 2A) (37). During the Iron Age and Persian periods (ca. 8th to 4th century BCE), it sequentially served as the residence of the local representative of the Assyrian, Babylonian, and Persian empires, as a center for tax collection, and for diacritical feasting events (38). From this site, we examined four storage jars, typical of the Judean region during the early Persian period (Fig. 2B), all found in a refuse pit, which contained mead according to previous organic residue analyses (37). One of these potsherds yielded a yeast strain designated RRPrTmd13.

In summary, we succeeded in isolating six yeast strains from 21 beer- and mead-related ancient vessels (Table 1 and Table S1).

### Negative controls.

One of the key questions in the current research is whether the yeast cells are descendants of the enriched ancient yeast cultures that fermented the liquid stored in the excavated vessels, or whether they are equally abundant in the environment. In order to answer this question, we used the above method to isolate yeast from non-beverage-related vessels and sediments from the surrounding environments of the excavated sites. To this end, we tested 27 samples from other ancient vessels from the same sites, vessels that were not associated with beverage storage but with other functions, including cooking pots, jugs and juglets, lamps, and bowls. None of these vessels yielded yeast (Table S1), save for the lamps (described below). Furthermore, we tested 53 samples of sediments and stones gathered from these archaeological sites, adjacent to the locations where the putative beverage vessels were found. These samples yielded two yeast strains: one was from a stone from En-Besor (Table S1, sample 26) which was identified by internal transcribed spacer (ITS) analysis as the pathogen Candida albicans (see Table S2 in the supplemental material) and is presumably a contamination originating from humans. The other one was from a sediment sample from Tell es-Safi/Gath (Table S1, sample 103), has an unidentified ITS, and is probably an undescribed wild yeast. Last, since yeast is often associated with plants (39), we also tested 30 samples of sediment and stones from the non-archaeological site Ma'on, as well as agricultural fields in the proximity of the sites of Tell es-Safi/Gath and Ramat Rachel. We were unable to isolate any yeast strains from these samples using our pipeline (Table S1). Overall, we found two yeast strains out of 110 non-beverage-related control samples. Thus, the findings of six yeast strains from 21 samples of putative fermented beverage vessels versus two yeast strains from 110 control samples is significant and hardly incidental (with a Fisher's exact test *P* value of 0.0006).

10.1128/mBio.00388-19.6 TABLE S2 Identification of yeast isolated in this work by ITS analysis and their ability to produce drinkable beer. Analysis was performed in the Isham barcoding database (http://its.mycologylab.org) and BLAST (https://blast.ncbi.nlm.nih.gov/Blast.cgi) with the ITS fragment of each yeast, which was amplified by PCR and sequenced (Sanger sequencing). In the “Drinkable beer production” column, “+” denotes drinkable beer “+/−” beer with spoilage aroma, and “−” nondrinkable beer. Download Table S2, DOCX file, 0.1 MB. Copyright © 2019 Aouizerat et al. 2019 Aouizerat et al. This content is distributed under the terms of the Creative Commons Attribution 4.0 International license.

### Genome sequencing of the isolated yeast.

While the ITS1 region was directly amplified and sequenced for all the isolates using PCR (Table S2), we sequenced the full genomes of the six yeast strains that had been isolated from beverage-associated ancient vessels. (For accession numbers, see Table 1.) We also sequenced the genome of one of the yeast strains that was isolated from the controls, RRPrNerP7 (accession no. SAMN08918674), which was isolated from an oil lamp found at Ramat-Rachel (see Fig. S1 in the supplemental material). This genome data was used to extract the genetic barcode large subunit (LSU) rRNA gene, to corroborate the ITS1 results, and to further carry out a full-genome BLAST analysis and phylogenomic analysis. The genomic data set was also used to investigate gene ortholog copy number variation (CNV) to shed light on the biochemical activity of the yeast, as described below. All yeast strains were identified based on similarities to yeast strain genomes from the NCBI database (see Fig. S2 in the supplemental material), and there was a match between the ITS identification and the full-genome sequencing.

10.1128/mBio.00388-19.1 FIG S1 Yeast from oil. (A) A schematic drawing of the Persian oil lamp excavated in a pit hole in the Ramat Rachel site, which yielded the yeast strain RRPrNerP7. (B and C) Yeast strain RRPrNerP7 cells (B) and colonies (C). (D) Colonies of yeast from sediments of 2-year-old bottle of olive oil demonstrating that yeast survive in olive oil. Download FIG S1, TIF file, 1.1 MB. Copyright © 2019 Aouizerat et al. 2019 Aouizerat et al. This content is distributed under the terms of the Creative Commons Attribution 4.0 International license.

10.1128/mBio.00388-19.2 FIG S2 Whole-genome BLAST analysis of the yeast strains isolated from ancient vessels. A BLAST analysis using full scaffolds as query and the NCBI online BLASTn database (https://blast.ncbi.nlm.nih.gov/Blast.cgi) as target was carried out for each yeast strain genome assembly. The bar plot depicts the target taxonomies that occurred in more than 5% of the hits. Download FIG S2, TIF file, 0.9 MB. Copyright © 2019 Aouizerat et al. 2019 Aouizerat et al. This content is distributed under the terms of the Creative Commons Attribution 4.0 International license.

**FIG 3:**
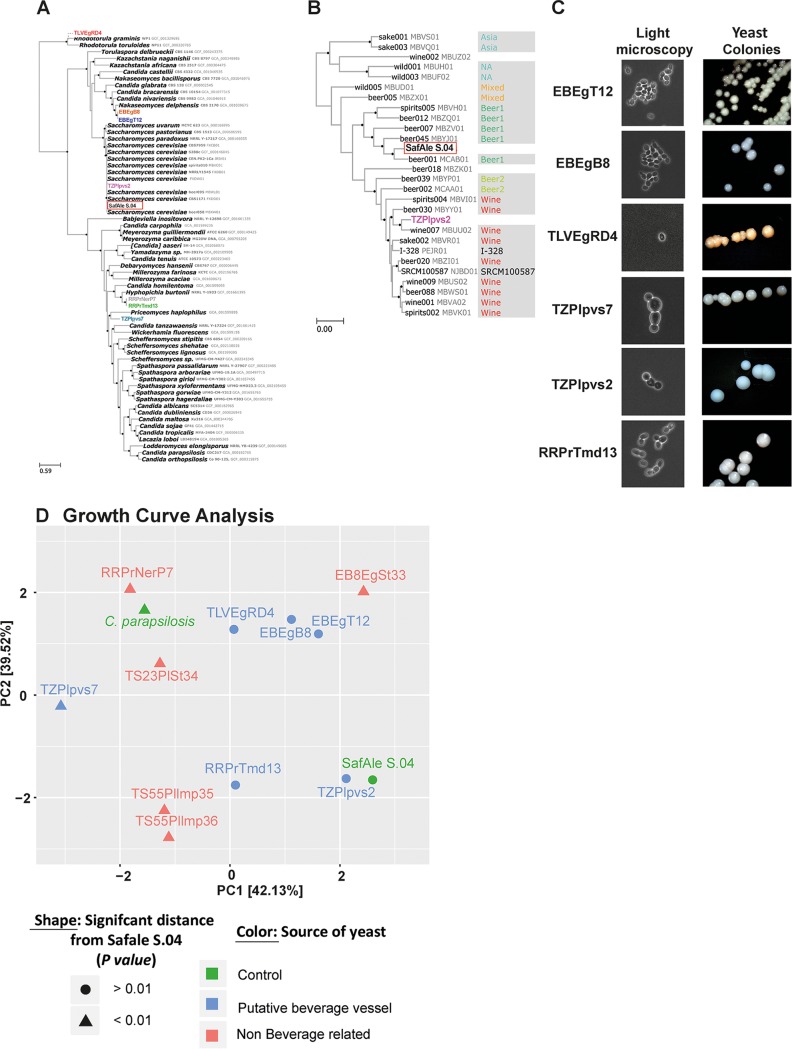
Genotypic and phenotypic characterization of yeast strains isolated from ancient vessels. (A and B) Phylogenetic trees based on full-genome sequencing of the isolated yeast strains. Black bullets at nodes represent maximal bootstrap percentage of node support. The newly isolated strains are in color. The modern beer yeast species Saccharomyces cerevisiae (SafAle S.04), which served as a control, is surrounded by a red box. (A) A list of 118 gene partitions and a representation of the combination *Saccharomycetaceae* plus *Debaryomycetaceae*. (B) Comparison of TZPlpvs2 (in purple) to modern wine and beer strains, based on 465 gene representatives and partitioning of Saccharomyces cerevisiae. Reference strains are denoted by NCBI strain name and accession number followed by clade affiliation (41). (C) The shape of the isolated yeast cells under light microscopy (left panel) and colonies on YPD agar plates (right panel). (D) Growth curve analysis. The yeast strain isolated from putative beverage vessels grows in beer wort with similar kinetics to a modern beer yeast. Principal-component analysis (PCA) of the distances of the growth curves of yeast grown in beer wort under fermentation-related conditions was performed (Fig. S3). The modern domesticated beer yeast strain SafAle S.04 served as a positive control, and the pathogenic yeast C. parapsilosis served as a negative control. The marker’s shape denotes the statistical significance of the distance from SafAle S.04 growth curve kinetics, and the color denotes the source of the yeast: control, putative beverage container, or non-beverage-related vessel.

The two yeast strains EBEgT12 and EBEgB8, which were isolated from the Egyptian vessels excavated at En-Besor, are genetically close to one another and show high similarities to Nakaseomyces delphensis (also known as Saccharomyces delphensis) (Fig. 3A and Table 1), which was isolated from dry African figs and is not common in soil (40). This supports the notion that the yeast cells originated from the vessels themselves, and not the environment, and suggests that perhaps figs were used in the fermented beverage production. Additionally, based on LSU rRNA barcoding analysis, these strains appear to belong to an unrecorded species (Table 1 and the supplemental material). To draw functional insight from the genomes of EBEgT12 and EBEgB8, we identified 596 orthologous gene clusters with copy number variation between the two isolates. Of these genes, we further compared 79 orthologous gene clusters of genes that were related to transmembrane transport and metabolism of various carbohydrates and were previously described as having copy number variations in beer-producing yeast strains (41). Despite the overall high genetic similarities between these two yeast strains (Fig. 3A), EBEgT12 had 67 genes with the expected duplications or deletions characteristic of beer yeast strains (see Table S4 in the supplemental material), whereas only 12 occurred in EBEgB8, which did not produce drinkable beer on its own (see below). This result significantly differs from the expected neutral result (*χ*^2^ = 21.9, *P* < 0.0001), suggesting that EBEgT12 was better adapted for beer production than EBEgB8, as indeed was observed while producing beer from these yeast strains (see the section on beer production).

10.1128/mBio.00388-19.8 TABLE S4 Comparison of duplicated genes related to wine production in the two genetically similar yeast strains isolated from Ramat Rachel. RRPrTmd13 was isolated from a mead vessel and produced drinkable beer, while RRPrNerP7, which was isolated from an oil lamp, did not. Presented here are the copy numbers of genes that are expected to have undergone duplication or deletion of copies in wine-producing yeast strains (41). The GO term and its description are provided using the eggNOG (49) database of orthologous groups and functional annotation (http://eggnogdb.embl.de/#/app/home). Download Table S4, DOCX file, 0.1 MB. Copyright © 2019 Aouizerat et al. 2019 Aouizerat et al. This content is distributed under the terms of the Creative Commons Attribution 4.0 International license.

10.1128/mBio.00388-19.3 FIG S3 Growth curves of yeast strains in beer wort. (A) Yeast cells were incubated in beer wort, and their growth was determined by recording the optical density (OD) of the culture at 600 nm. Dotted lines denote the positive and negative controls: i.e., the modern beer yeast SafAle S.04 and the pathogen C. parapsilosis, respectively. Solid lines denote yeast strains that were isolated from putative beverage-containing vessels, and dashed lines are yeast strains from non-beverage-related vessels, lamps, soil, stones, and sediments. Each line is an average of at least 3 independent colonies. (B) For comparison, the data from each yeast strain were fitted to a logistic equation. The numbers denote the *P* value of the differences of the growth rate parameter (*r*) of the fitted equation between the growth curve of SafAle S.04 and the curve of each yeast strain. In blue are *P* values of >0.01 (similar to SafAle S.04), and in red are *P* values of <0.02 (far from SafAle S.04). See also Fig. 3 for principal-component analysis (PCA) of these data demonstrating the relative distances between the curves. Download FIG S3, TIF file, 1.4 MB. Copyright © 2019 Aouizerat et al. 2019 Aouizerat et al. This content is distributed under the terms of the Creative Commons Attribution 4.0 International license.

TLVEgRD4, the third yeast strain that was isolated from the Egyptian vessels, showed high similarity (Fig. 3A and Table 1) to the red-pigmented yeast Rhodotorula glutinis, which is a known food-contaminating agent found in Nigerian and other beers (42, 43).

From one of the Philistine vessels, we isolated yeast strain TZPlpvs7, which was found to be similar to yeast strains of the *Debaryomycetaceae* family (Fig. 3A and Table 1). Members of this family were isolated from traditional African beers brewed with sorghum malt (44, 45). The second yeast strain isolated from the other Philistine vessel, TZPlpvs2, is Saccharomyces cerevisiae, which is the most commonly used species of domesticated yeast and plays a central role in modern beer, wine, and bread industries (3). We further tested the similarity of TZPlpvs2 to known beer- and wine-producing S. cerevisiae strains and found that it is close to strain Wine-007 (NCBI assembly MBUU02 [Fig. 3B]), a modern yeast strain used in wine production (41).

RRPrTmd13 which was isolated from a mead-containing vessel (based on organic residue analysis), was found to be similar to the yeast Hyphopichia burtonii (Endomycopsis burtonii) (Fig. 3A and Table 1). Significantly, this yeast species was previously isolated (22, 46) from tej, an Ethiopian honey wine (47)—a type of traditional African mead.

We also sequenced the genome of the yeast strain RRPrNerP7 (NCBI accession no. SAMN08918674), which was isolated from a clay oil lamp (Fig. S1) from Ramat-Rachel. Surprisingly, its sequence was found to be similar to that of Hyphopichia burtonii (Fig. 3A), like RRPrTmd13, the mead vessel yeast strain that was isolated from the same site. Nevertheless, RRPrNerP7 and RRPrTmd13 were divergent from each other in phenotypes related to several beverage production aspects. We compared RRPrTmd13 and RRPrNerP7 regarding duplications and deletions in 52 orthology clusters with gene ontologies related to the metabolism of various carbohydrates and the transmembrane transport of iron, sodium, and sugars, found to have characteristic copy number variations in modern wine-producing S. cerevisiae yeast (41), although not specifically studied in mead-producing yeast strains. As can be seen in Table S4, the duplications and deletions in both isolates occurred in significantly different orthology clusters (*t* test, *P* = 2 × 10^−7^). Additional phenotypic differences between RRPrTmd13 and RRPrNerP7 are described below.

### Genome-wide BLASTn.

Each genome assembly was analyzed with the online version of BLASTn. To summarize the results, we considered all the taxonomic IDs that constituted more than 5% of the matches (Table 1 and Fig. S2). For isolate TZPlpvs7, over 50% of the scaffolds matched to one of three debaryomycetacid species, with only 80% identity (standard deviation [SD], 4.1%) to all the species, and over 40% of additional scaffolds matched debaryomycetacid species with lower identities. Similarly, the best match of almost all the scaffolds of isolates RRPrTmd13 and RRPrNerP7 was *H. burtonii*, albeit with a mean percentage of identity of only 84.5% (SD, 4.5%). Finally, the best matches of 50% and 97% of the scaffolds of isolates EBEgB8 and EBEgT12, respectively, were in the *Nakaseomyces*/*Candida* clade, but with a low mean percentage of identity of only 83% (SD, 6%). We would thus suggest that these five isolates represent species that are not yet recorded in the NCBI nucleotide repository. Conversely, for TZPlpvs2, over 60% of the scaffolds had 99.9% identity (SD, 0.14%) with S. cerevisiae, indicating that this isolate is very similar to records of S.cerevisiae, in agreement with the phylogenomic analysis.

### Phylogenomic analysis.

To validate the phylogenetic position of the isolates, we selected reference genome assemblies of 55 isolates that are available on GenBank (see Fig. 3A for accession numbers). We then annotated coding sequences in the reference genomes as well as in our isolate genome assemblies, using Augustus 3.2.3 (48). For the annotation process, we chose the coding sequences of the nearest available reference relative as hints (Fig. 3A) and either *Saccharomyces* or Candida tropicalis as the model species for *Saccharomycetaceae* and *Debaryomycetaceae* species, respectively. We extracted a protein sequence file for each isolate genome and reference genome and assigned orthology information to each gene with eggNOG 4.5.1 (49; http://eggnogdb.embl.de/#/app/home). We selected orthologs with one representative in at least 50% of the reference genomes and in at least three out of our five isolates. Protein sequences of each ortholog were aligned with MAFFT (50) using the L-ins-i algorithm, and each ortholog alignment was trimmed with TrimAl using the gappyout algorithm. Using treeCl (51), we reconstructed maximum likelihood gene trees for each ortholog and clustered the resulting gene trees based on the weighted Robinson folds (WRF) (52) pairwise intertree distances and the db-scan clustering algorithm, to assess the existence of conflicting phylogenetic signals. For every cluster, treeCl produces a supermatrix of all the genes in the cluster, which we used for a partitioned tree reconstruction with RAxML (53) using the LG evolutionary model and 100 thorough bootstrap replicates for branch support of Saccharomyces cerevisiae. To recover the phylogenetic position of two S. cerevisiae isolates (SafAle S04 and TZPlpvs2), we repeated the workflow described above, using a targeted reference data set of 26 S. cerevisiae genomes covering the diversity of known isolates, as described by Gallone et al. (41). In this case, we retained one to one orthologs represented in at least 70% of the reference genomes and in both of our isolates. Due to the high sequence identity among the analyzed genomes, all belonging to S. cerevisiae, we retained only the most informative 650 orthologs by selecting alignments with at least 10 unique sequences and at least 10 parsimony informative alignment columns (i.e., at least two character states in the column, each occurring in at least two sequences).

The sequence alignments of 118 orthologs passed our filters and were included in the analysis of the data set *Saccharomycetaceae* plus *Debaryomycetaceae*. Conflicting phylogenetic signals were not detected among them, as the db-scan algorithm has detected only one cluster, which was robust to changes in minimal local radius cutoff. The phylogenetic tree was reconstructed from a supermatrix of all 118 orthologs (Fig. 3A) with the matrix and partition (uploaded files 6 and 7, Table S7). Isolates EBEgT12 and EBEgB8 were very similar with less than 4 × 10^−4^ substitutions per base (SPB). They clustered as the sister clade of Nakaseomyces delphensis, but with much larger sequence divergence (over 0.13 SPB). Isolate TZPlpvs7 was resolved as a *Debaryomycetaceae* sp., which is divergent from other confamilials for which a genome assembly is available (at least 0.8 SPB). Isolates RRPrTmd13 and RRPrNerP7 were very closely related to each other (less than 4 × 10^−4^ SPB) and emerged as a sister clade of *Hyphopichia burtonii* (*Debaryomycetaceae*) with a sequence divergence of over 0.2 SPB. Isolate TZPlpvs2 clustered within the S. cerevisiae clade (Fig. 3A) with maximal node support. Based on our S. cerevisiae*-*focused phylogenomic analysis (Fig. 3B), TZPlpvs2 is a part of the “Wine” cluster as was recovered by Gallone et al. (41). This cluster originally included both beer and wine yeasts. It is most closely related to isolate “Wine-007” with maximal node support, and with sequence divergence of 8 × 10^−4^ SPB. This sequence divergence is larger than those observed between RRPrTmd13 and RRPrNerP7 or between EBEgT12 and EBEgB8 and is not contrary to observed phenotypic differences. The S. cerevisiae*-*focused analysis included 650 orthology clusters that passed the filtering steps (see Materials and Methods). In this analysis, the number of gene tree clusters was maximized when using a minimal local radius of 0.03 in the db-scan analysis and resulted in two tree groups of 465 and 185 trees. Figure 3B is based on the larger group, whereas the smaller group yielded a tree with a similar topology, but with an overall shorter tree distance. It is thus an artifact of the weighting procedure of the WRF parameter and does not represent a real phylogenetic conflict. As we observed different phenotypes in isolates EBEgT12 and EBEgB8, with only isolate EBEgT12 producing beer, we expected that this difference will be reflected in gene copy number variation (CNV) between the beer-producing yeast and the non-producing yeast, as previously shown by Gallone et al. (41). Despite the overall high genetic similarities between these two yeast strains, we identified 79 orthology clusters with CNV between the two isolates, which were related to transmembrane transport and metabolism of various carbohydrates, also described by Gallone et al. (41) as having CNV in beer-producing yeasts. EBEgT12 had 67 genes with the expected duplications or deletions in beer yeasts (Table S4), while EBEgB8 had only 12, supporting EBEgT12 as better adapted for beer production than EBEgB8.

10.1128/mBio.00388-19.7 TABLE S7 Uploaded additional files 1-11. Genetic sequence analysis input and output files were uploaded and are available on FigShare (https://figshare.com), with DOI numbers as provided in the following legends. Download Table S7, DOCX file, 0.1 MB. Copyright © 2019 Aouizerat et al. 2019 Aouizerat et al. This content is distributed under the terms of the Creative Commons Attribution 4.0 International license.

Additionally, we observed different phenotypes in isolates RRPrTmd13 and RRPrNerP7, with only isolate RRPrTmd13 producing mead. In this case, we also expected that this difference would be reflected by CNV instances between the two isolates. Although Gallone et al. (41) did not analyze mead-producing yeasts, wine shares some of the sugar sources with honey-based mead, and similarly, the mead-producing isolate (RRPrTmd13) shares some of the duplications and deletions with the wine-producing isolate (41) compared with isolate RRPrNerP7 (Table S1). In this case, however, both isolates had similar numbers of the CNV instances expected in wine yeasts (27 and 25 for RRPrTmd13 and RRPrNerP7, respectively), providing no prediction as to the expected phenotype.

### Taxonomic identity of isolates based on LSU rRNA barcoding.

Taxonomic classification of isolates EBEgT12 and TLVEgRD4 was assessed via LSU rRNA barcoding, as this marker has been comprehensively sampled across the taxonomy of Ascomycota, and is more variable than the small subunit (SSU) rRNA gene (54). BLASTn 2.6.0+ (55) was used to identify the LSU rRNA locus in each genome assembly, with the LSU rRNA SILVA (54) database sequences as query and the genome assemblies as target. In each genome assembly, the best match to any of the target sequences in each assembly was recovered as the isolate’s LSU rRNA gene. We further composed a relevant reference data set by running a second BLASTn analysis, in which the isolate LSU rRNA sequences were used as queries and the LSU rRNA SILVA database as target. The best 500 matches to each of the isolate sequences were retained, and redundancies were eliminated by retaining only the centroid sequences of 99% identical clusters, as predicted with VSEARCH v2.4.3 (56). The resulting data set, together with the isolate LSU rRNA sequences recovered from the genome assemblies, was used in a phylogenetic analysis to identify the phylogenetic position of the isolates. The sequences were aligned with MAFFT v7.310 (50), positions with over 0.8-gap proportion were removed with TrimAl v1.4.rev15 (57), and a phylogenetic tree was built with RAxML 8.2.10 (53), using the GTRGAMMA model and 100 replicates of rapid bootstrap trees for node support.

To check whether the isolates belonged to established species, we calculated all the intraspecies patristic distances (the cumulative branch length between two tree nodes) in the LSU rRNA phylogenetic tree and computed their distribution. We then calculated the patristic distance between each isolate for which LSU rRNA was recovered and its closest relative and tested whether this distance belonged to the distribution of the intraspecific patristic distances. Patristic distances were computed with ETE 3 (58). Our trimmed nonredundant LSU rRNA sequence alignment included 350 reference sequences and the isolates EBEgT12 and TLVEgRD4 (Table 1), with 853 positions and less than 0.1% missing data. The redundant data set, as well as the nonredundant alignment and the trimmed alignment, is included (uploaded files 1 to 4, Table S7). The maximum intraspecific patristic distance in our resulting tree was 0.012 substitution per base (SPB). Only isolate EBEgT12 was divergent enough from its closest relative (Nakaseomyces delphensis; *Saccharomycetaceae*) to constitute a novel species (0.023; *P* = 0.02). It is worth noting that by removing redundant sequences, we overestimated the *P* value, and this result is thus very conservative. Isolate TLVEgRD4 was found to be identical to *Rhodotorula glutinis* (*Sporidiobolaceae*; 2 × 10^−6^ SPB). The LSU rRNA phylogenetic tree is in uploaded file 5. The results of all the analyses are summarized in Table 1.

### Phenotypic characterization of the isolated yeast.

We compared several phenotypes of the isolated yeast strains related to alcoholic beverage production. As a positive control, we used the modern, commercially available beer yeast strain S. cerevisiae SafAle S.04 (Fermentis Division of S.I. Lesaffre, Marcq-en-Baroeul, France). First we compared the morphology of cells and colonies (Fig. 3C, left panel), using phase light microscopy to image colonies (Fig. 3C, right panel) on agar plates containing the lab standard yeast medium YPD (yeast extract-peptone-dextrose). All yeast strains showed the common structure of budding yeast cells and white smooth colonies, with the exception of TLVEgRD4, which yielded red colonies. The red pigmentation is in agreement with its identification as *R. glutinis*, which produces several carotenes, including β-carotene (59).

Next, we hypothesized that the isolated yeast strains were naturally selected to grow under beverage fermentation conditions and would be able to grow in beer wort, similar to modern domesticated beer yeast strains. To test this hypothesis, we compared growth kinetics of ancient isolated yeast strains to those of the modern beer yeast strain SafAle S.04 when grown in wort (see Fig. S3A in the supplemental material). As a negative control, we used the pathogenic yeast species Candida parapsilosis (60), which, unquestionably, is not used for beverage production. To compare the growth curves, we fit each curve to a logistic equation (Fig. S3B) that models growth curves (61). Next, we calculated the relative distance between the various fitted equations using principal-component analysis (PCA), demonstrating the relative similarities between the parameters of the fitted curves (Fig. 3D). We found that a high correlation (*r* = 0.95) exists between the growth curve shape and whether the yeast strain was isolated from a putative beer vessel or not. All yeast strains isolated from vessels that were believed to have originally contained fermented beverages grew similarly to SafAle S.04, except for TZPlpvs7, while all the other yeast strains, from lamps, sediments, and stones, showed different growth kinetics than SafAle S.04 (Fig. 3D). These results suggest that indeed the yeast strains isolated from the putative beverage containers are progenies of yeast that were selected in the past for growth under fermentation-related conditions.

### Analysis of beer produced by the isolated yeast.

Finally, as supportive evidence for their identity, we tested the ability of the isolated yeast strains to produce drinkable alcoholic beverages. To this end, we performed an initial screen using a standard common recipe of beer brewing (62) with each one of the isolated yeast strains. Strains EBEgT12, TZPlpvs2, and RRPrTmd13 produced aromatic and flavorful beer and were taken for additional compound and flavor analyses. Strain TZPlpvs7 produced beer that was drinkable but had a slight spoiled off-taste. In contrast, the following yeast strains were excluded from further analysis: EBEgB8, and the yeasts EB8EgSt33 and TS23PlSt34, which were found in stones, and the yeasts isolated from oil lamps, RRPrNerP7, TS55Pllmp35, and TS55Pllmp36, which produced beer with mild or strong spoiled aroma and flavors (63). TLVEgRD4 was also excluded, as *Rhodotorula glutinis* was reported to be a pathogenic beer spoiler yeast species (64).

**FIG 4:**
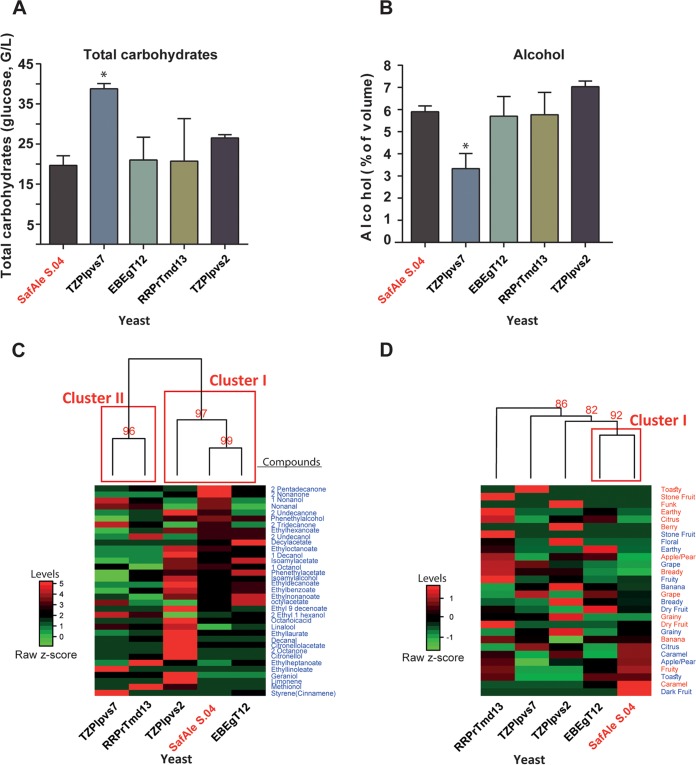
Characterization of reconstructed beer produced by yeast strains isolated from ancient vessels. Beer was brewed with the yeast strains isolated according to a standard brewing recipe. The modern beer yeast strain S. cerevisiae (SafAle S.04) served as a positive control. (A) Levels of total carbohydrates in the beers (as glucose). (B) Amount of alcohol produced. (C) Heat map of clustered levels of aromatic and flavor compounds found in the beer. The levels of various compounds were normalized as a percentage of the highest value for each compound. (D) Heat map of clustered samples based on parameters of beer tasting of aromas (red text) and flavors (blue text) (see also Fig. S4 in the supplemental material). In both panels C and D, the clustering was performed using Ward’s method with Euclidean distances. In the red text are the approximated unbiased (AU) *P* values in percentages of the nodes. The red squares denote clusters with *P* values of *>*95%.

Next, we compared the beer produced by the yeast, which passed the initial screening, to that produced by the positive control, to SafAle S.04 (Fermentis Division of S.I. Lesaffre, France). Comparison of the total carbohydrate (Fig. 4A) and alcohol (Fig. 4B) concentrations produced by yeast showed that besides TZPlpvs7, all the other yeast strains exploit carbohydrates and produced about 6% alcohol, similar to the “professional” beer yeast strain SafAle S.04.

10.1128/mBio.00388-19.4 FIG S4 Taste analysis of aroma and flavors of beers produced by the yeast strains isolated from ancient vessels. See also Fig. 4D for a heat map of these results. Download FIG S4, TIF file, 1.3 MB. Copyright © 2019 Aouizerat et al. 2019 Aouizerat et al. This content is distributed under the terms of the Creative Commons Attribution 4.0 International license.

We performed further qualitative analyses of several aromatic and flavor compounds in the various beers by headspace solid-phase microextraction–gas chromatography-mass spectrometry (HS-SPME-GC-MS) (Table S6). The detected compounds were either known to be present in beers (65–67) or are other members of the alcohol, ester, monoterpenoid, and carboxylic acid groups. This analysis shows that relatively high ratios of many aroma compounds were detected in the beer produced using the TZPlpvs2 strain, which was no surprise, as this strain was identified as S. cerevisiae. Moreover, comprehensive analysis of aromatic and flavor compounds (Table S6) shows that TZPlpvs2 and EBEgT12 produced beers that clustered with SafAle S.04 (Fig. 4C).

10.1128/mBio.00388-19.7 TABLE S6 Qualitative analysis of aromatic and flavor compounds in beers produced by yeast strains from ancient vessels. Qualitative analysis of relative peak areas for each compound across our samples was conducted using SPME Fiber (DVB/CAR/PDMS), followed by separation by gas chromatographer and detection by Agilent 5973 mass spectrometer (MS) detector in Full-Scan mode. Suggestions for the identification of the detected peaks were carried out by Wiley mass spectrometry database. Semi-quantitative analysis was based on the relative peak areas calculated using the integration order in the ChemStation Software and using the peak area of ethanol as an internal standard. Ethanol quantities in each sample were determined by distillation, and each peak was normalized in accord with the relevant ethanol quantity in each sample. The relative peak area for each compound was calculated by dividing the peak area of the compound to that of the normalized ethanol peak area, and multiplied by 1000, to get more presentable numbers. Download Table S6, DOCX file, 0.1 MB. Copyright © 2019 Aouizerat et al. 2019 Aouizerat et al. This content is distributed under the terms of the Creative Commons Attribution 4.0 International license.

The beers that passed the initial screen were also compared by organoleptic descriptive analyses performed by members of the Beer Judge Certification Program (BJCP [https://www.bjcp.org]), a beer taster’s organization. The results of these analyses were in agreement with the chemical analysis: RRPrTmd13 and, to a lesser extent, TZPlpvs2 produced beers that are similar in color, aroma, flocculation, and flavor to that of SafAle S.04 (Fig. 4D and Fig. S3).

In summary, these results exemplify the potential in the research of live microorganisms isolated from ancient vessels and their products. Nevertheless, it should be noted that aromatic beer can be produced also by wild yeasts (68) and even pathogenic yeasts such as C. parapsilosis (69), and thus beer production *per se* cannot support or reject the hypothesis that the isolated yeast are descendants of the fermenting yeast.

### Ancient lamps.

An exception to the notion that yeast can be significantly better isolated from ancient beverage containers in comparison to other vessels was clay lamps, which usually contained olive oil, from both Tell es-Safi/Gath and Ramat-Rachel. Surprisingly, we succeeded in isolating three yeast strains from six ancient lamps (Fig. S1A to C). A possible explanation to the presence of yeast in these lamps might be that the yeast derives from yeast cells existing on olives. These yeast cells were not killed during the cold-press extraction of the oil and were absorbed into the pores of the ceramic matrix of the lamps. This is in agreement with previous observations showing that yeast are the predominant microorganisms in olive oil (70, 71) and their concentration ranges between 10^3^ and 10^5^ cells/ml (72). We also confirmed that yeast cells are indeed present in olive oil by isolating live yeast cells from a modern bottle of olive oil that had been sealed for 2 years (Fig. S1D). Finally, we identified the yeast strains by amplification and sequencing of their ITS region and found that the two yeast isolates from the vessels from Tell es-Safi/Gath are strains of Yarrowia lipolytica (TS55Pllmp35 and TS55Pllmp36 [Table S2]), a yeast strongly associated with oil flora (73, 74), which is not used for beer production (75). Thus, we suggest that the oil lamp results support the notion that yeast colonies remain alive in ancient clay vessels and it is feasible to isolate them.

## DISCUSSION

In this study, we isolated yeast cells from ancient vessels excavated at archaeological sites in Israel. These vessels belong to vessel types that, based on their shape, or, in the case of the vessel from Ramat Rachel, based on organic residue analysis, were considered to have contained fermented beverages such as beer and mead (honey wine).

The main challenge of this research lies in the question of whether the isolated yeast strains originated from the ancient yeast that fermented ancient beverages in the archaeological vessels and whether the yeast cells that were discovered are in fact descendants of the original yeasts, having survived and continued to grow in microenvironments in pores within the clay matrix of the vessels. Or, perhaps, they are wild yeast from the environment or a recent contamination. Several lines of evidence strongly suggest that the yeast strains we isolated are indeed descendants of fermenting yeasts in the ancient vessels:

First, the number of isolated yeast strains from putative beverage vessels (6 out of 21 samples), in comparison with the yeast strains isolated from the control samples (2 out of 110 samples), is significantly biased toward the beverage-related vessels;

Second, all yeast strains isolated from the putative beverage vessels besides TZPlpvs7 grew in beer wort medium, similar to the modern domesticated beer yeast SafAle S.04, while all the yeast strains isolated from the control samples show different growth parameters under these conditions (Fig. 3).

Third, the molecular phylogeny of the yeast strains isolated from the ancient vessels also supports the notion that they originated from ancient, fermented-liquid-related yeast strains. TZPlpvs2, being S. cerevisiae, the major fermenting yeast species today (76), is often found on fruits and flowers and less in soil (77). EBEgT12, EBEgB8, and TZPlpvs7 are similar to yeast species found in various traditional beverages in Africa (44, 45). Yeast strain RRPrTmd13, which was isolated from a mead (honey wine) vessel, identified as such through organic residue analysis, is highly similar to a yeast species found in the Ethiopian honey wine tej (46). Lastly, the two yeast strains TS55Pllmp35 and TS55Pllmp36, isolated from Philistine oil lamps, were found to be distinct strains of Yarrowia lipolytica, a yeast that tends to grow in olive oil (73, 74). In contrast, the only two yeast strains isolated from a stone and sediment control sample were C. albicans—probably from human contamination—and an unidentified yeast, respectively, both of which did not produce beer.

Fourth, it is improbable that these yeast cells originated from sediments or from handling contaminations. At least in the case of Nakaseomyces delphensis, the closest yeast species to the two isolated yeast strains EBEgT12 and EBEgB8 was reported to be found on figs and rarely in soil (40). Furthermore, the possibility that the source of the yeast cells is a contamination from modern beer is unlikely, since besides the S. cerevisiae yeast (TZPlpvs2), all the other isolated yeasts are not commonly used in the modern beer industry and thus could not have derived from modern unfiltered beer. It should also be noted that although TZPlpvs2 is S. cerevisiae, its sequence is clearly different from those of commonly used S. cerevisiae laboratory strains, further excluding the possibility of contamination in the lab.

Fifth, the observation that most of the yeast strains isolated from putative beverage containers produced drinkable aromatic and flavored beer (Fig. 4), while all the control isolated yeast strains produced spoiled aromas and flavors, also provides some support for the authenticity of the origin of the isolated yeast as descendants of the ancient fermenting yeast. As mentioned above, this support is weaker than the previous ones, because some wild yeast can also ferment beer and produced aromatic compounds (68, 69). Thus, there is a correlation between the source of isolation (putative beverage vessel or not), similar growth in wort compared to modern beer yeast strains, and the ability to produce drinkable beer. Such a correlation suggests that the yeast strains isolated from putative beverage vessels are descendants of yeast strains that have experienced the selection pressures of alcoholic fermentation and beverage production environments and continued to reproduce over the ages in microenvironments within the ceramic matrices of the ancient beverage vessels.

Taken together, we suggest that the evidence strongly supports the authenticity of the yeast strains isolated from ancient vessels as ancient beverage yeast. We assume that the large amounts of yeast cells that grew during repeated series of fermentations in these vessels, in antiquity, were absorbed into the nanopores of the vessels. These yeast cells altered the composition of the microorganisms’ population and remained as microcolonies, which continued to grow and survive over millennia in the ceramic matrices, based on occasional supply of moisture and nutrients. This assumption is also based on the observation that yeast cells survived in a clay vessel that was buried for 3 weeks and a clay jug that was exposed to the hot and dry weather of Israel for 2 years (Fig. 1A). Additional support of this assumption is the well-known fact that in many traditional beer production methods, it is common to use the residues within vessels to serve as “starters” for the production of the next batch of fermented food. This technique is described in ancient inscriptions (4) and is still used in modern traditional beer-brewing techniques (78), as well as for the production of wine (79), yoghurt (80), and bread (81). Practically speaking, the ancient producers, using selection processes, domesticated yeast and bacteria that produced “good” and tasty fermented food, similar to the selection processes in the domestication of plants and animals. This perhaps could explain the findings that EBEgT12 and EBEgB8, isolated from Egyptian vessels from En-Besor, show high genetic similarities to each other, yet they differ in several of the hallmark genes typical of beer-producing yeast strains, which seem to be mirrored in the quality of the beer that they produced. While EBEgT12 beer contained aromatic and flavor compounds, the beer made from yeast strain EBEgB8 had mildly spoiled aroma and flavors. Possibly, both of them were included in the original brew, complementing each other, or maybe EBEgB8 represents the undomesticated ancestor of EBEgT12, before it was selected for “good” beer making, as in the case of the domestication process of S. cerevisiae (76). These questions may be answered by additional isolation and analysis of yeast strains from more beverage-containing vessels, which will shed further light on the yeast domestication processes.

In addition, the two yeast strains isolated from the Persian period vessels, RRPrTmd13 from the mead container and RRPrNerP7 from an oil lamp, show overall high similarity to each other, including similar genes associated with wine production (see Table S3 in the supplemental material). However, they diverge in the orthology clusters that have experienced CNV, in growth under fermenting conditions, and in the quality of the beer they produced. In this case, we speculate that RRPrNerP7 represents the wild yeast ancestor, which naturally resides on olives (82, 83), while RRPrTmd13 is a domesticated descendant, which was selected for “successful” mead production. It might also suggest that wine and oil were prepared at proximal sites.

10.1128/mBio.00388-19.7 TABLE S3 Comparison of duplicated genes related to beer production in the genetically similar yet distinct beer-producing yeast strains EBEgT12 and EBEgB8, both found in En-Besor. Presented here are the copy numbers of genes that are expected to have undergone duplication or deletion of copies in beer-producing yeast strains (41). In addition, we present the Gene Ontology (GO) term and its description using the eggNOG ortholog database of orthologous groups and functional annotation (http://eggnogdb.embl.de/#/app/home) (49). Download Table S3, DOCX file, 0.1 MB. Copyright © 2019 Aouizerat et al. 2019 Aouizerat et al. This content is distributed under the terms of the Creative Commons Attribution 4.0 International license.

Regarding the red colony yeast TLVEgRD4 (*R. glutinis*), we suggest that it contaminated the ancient beverage, as happens today in modern traditional beers (42), or perhaps, although less likely, it was part of the beer sediments and contributed to its flavors.

In summary, based on all of the above findings, we propose that it is highly likely that yeast strains EBEgT12, RRPrTmd13, and TZPlpvs2 are the descendants of the original ancient beverage-producing yeast strains. We are less confident about TZPlpvs7, EBEgB8, and TLVEgRD4, which are perhaps descendants of contaminators of the ancient beverages. The yeast isolated from lamps originated, most probably, from yeast that grew in the oil, and the remaining yeast strains from sediments and stones are probably wild yeasts.

It should be noted that for comparative reasons, the beers were brewed for these analyses using single yeast strains only, with a standard modern recipe. It is possible that brewing beverages using traditional recipes, ingredients, and mixtures of the yeast strains and including those seemingly less fit for beverage production would have improved the brew quality. Moreover, it is highly likely that the yeast strains we isolated here represent only a portion of the rich variety of microorganisms that originally inhabited the vessels and contributed to the fermentation processes of the ancient beverages.

In conclusion, we demonstrate here that isolating, growing, and studying fermenting microorganisms from ancient vessels in order to expand archaeological knowledge of ancient diet and food-related technologies are feasible. These results, which allow a more precise recreation of ancient-like beverages than ever before, unlock enormous potential for the study of a broad range of food-related issues in antiquity. This includes expanding the knowledge about the ancient diet of diverse societies in many periods and locations, the study of the functions of ancient vessels, facilities, and infrastructures, understanding links between cultures or identity groups and technological transfer between them, uncovering trade routes and food preparation technologies, and even obtaining insights into the actual somatic aspects (aroma and flavors) of ancient foods and beverages.

Furthermore, the findings here might open new avenues in archaeological research, since we speculate that isolation of microorganisms from ancient remains is not limited to yeast, and it would be even easier to isolate bacteria due to their remarkable survival abilities. Thus, this kind of approach can most probably be expanded to a broad range of topics, from disease-borne bacteria to food-associated bacteria, such as those used in fermented beverages, cheese, and pickles.

The next steps of the research, currently conducted in our lab, will include “fingerprinting” of modern and ancient vessels that contained various kinds of fermented foods and liquids. This is performed using combined microbiome-like DNA analysis and microorganism’s isolation, which we believe will provide valuable data on the dating, identification, and characterization of food containers and ingredients and even the reconstruction of ancient diets.

## MATERIALS AND METHODS

### Yeast growth.

Unless otherwise mentioned, the yeast strains used in this work were routinely grown from a single colony, either in liquid YPD medium (Difco, USA) at 30°C under aerobic conditions with agitation (250 to 300 rpm) or on solid YPD medium containing 2% wt/vol Bacto agar (Difco, USA) incubated at 30°C. Stocks of yeast strains were kept in −80°C in 50% glycerol.

### Preparation of control modern vessels for yeast isolation.

A modern clay vessel was broken into equally sized and shaped pieces and divided into two groups: group A pieces were buried “as is,” in three pits that were 30 cm deep and with a 2-m space between them in a city garden. Group B shards were buried in the same way in a different city garden, situated several hundred meters away. Prior to covering them up, the pieces of group B were sprayed with 300 ml of unpasteurized lager beer with a vital colony of the branded strain Fermentis-WB-34/70. After 6 weeks, the pottery from both sites was retrieved and sent to the lab for yeast cell revival and isolation.

### Yeast isolation from vessels and control samples.

The vessels were entirely flooded with rich YPD medium (Difco, USA) and incubated at room temperature for 7 days. Then, samples from the medium were streaked on selective agar plates for fungal isolation (NOVAmed BA-114, Israel) and incubated at 30°C for 12 to 48 h. Yeast colonies growing on the plates were replated on solid YPD agar plates, containing 2% wt/vol Bacto agar (Difco, USA). Colonies were picked for further analysis.

### Electron microscopy.

Ceramic samples were cut using a diamond disc power cutter (Dremel). The surface morphology of the archaeological ceramic samples was examined using the FEI Quanta 200 scanning electron microscope situated in the core facility of the Hebrew University Medical School in Ein Kerem. Samples were first sputtered by Au/Pd (SC7620; Quorum Technologies). Images were then taken with a secondary electron detector at magnification ×10,000 to ×40,000 using a 10- to 30-kV accelerating voltage and a lens objective aperture of 30 to 20 μm.

### DNA purification.

Yeast cell DNA isolation was performed as previously described (84). Briefly, 10 ml of overnight cultures was centrifuged at 3,000 rpm for 5 min and washed in sterile water. The cells were treated with 200 μl of phenol chloroform, 0.3 g of acid-washed glass beads, and 200 μl of Smash and Grab solution (84) and lysed using a vortex for 3 min, after which TE buffer was added. The cells were centrifuged, and the aqueous layer containing the DNA was transferred to 1 ml ethanol and then washed and suspended in Tris-EDTA (TE) buffer. One microliter of RNase (10 mg/ml DNase- and protease-free RNase; Thermo Fisher Scientific) was added, and the solution was incubated at 37°C for 5 min. Ten microliters of ammonium acetate (4 M) and 1 ml of ethanol were then added, and the solution was washed, and suspended in 100 μl of TE buffer. The extracted DNA was stored at −20°C. DNA quantification was carried out on a Synergy H1 microplate reader (BioTek Instruments, Inc., VT), using a Take3 microvolume plate.

### ITS analysis.

The internal transcribed spacer (ITS) region of the yeast was amplified using standard Illumina primers as described at the Earth Microbiome Project website (http://www.earthmicrobiome.org/protocols-and-standards/its/). PCR fragments were Sanger sequenced by the interdepartmental sequencing unit of the Hebrew University. The sequences were identified by BLAST analysis against the ISHAM barcoding database (http://its.mycologylab.org) and the NCBI database (https://blast.ncbi.nlm.nih.gov/Blast.cgi).

### DNA sequencing.

Sequencing was performed in the interdepartmental unit at the Hebrew University, Hadassah Ein Karem Campus. Libraries were prepared by using a Nextera XT DNA kit (Illumina, San Diego, CA), and DNA was amplified by a limited-cycle PCR and purified using AMPure XP beads. The DNA libraries were normalized, pooled, and tagged in a common flow cell at 2 × 250 base-paired-end reads using the NextSeq platform.

### Genome assembly.

Illumina adaptors were removed with Trimmomatic 0.36 (85). The quality of the reads was determined using FastQC (https://www.bioinformatics.babraham.ac.uk/projects/fastqc/). *De novo* assembly was then carried out with the Celera assembler 8.3rc2 (86), and non-target-species scaffolds were excluded using BlobTools V1 (87). The sequencing and genome assembly effort was targeted at obtaining assemblies contiguous enough to derive protein coding gene data for phylogenomic analyses (Table S5). The resulting genome assemblies had coverages of 36× to 240× and *N*_50_ values of 2,842 to 15,623 bp (Table S5). These data provided us with at least 2,702 protein coding genes per sample with a median length of at least 294 amino acids (aa). The gene count variation could be the result of ploidy differences or of genome assembly artifacts and may cause the underestimation of one-to-one orthologs. However, we were still able to curate a large and high-quality one-to-one ortholog gene subset to perform the phylogenomic analyses.

10.1128/mBio.00388-19.7 TABLE S5 Genome assembly statistics. N50 denotes the median length of contigs. Download Table S5, DOCX file, 0.1 MB. Copyright © 2019 Aouizerat et al. 2019 Aouizerat et al. This content is distributed under the terms of the Creative Commons Attribution 4.0 International license.

### Beer preparation.

For beer production comparison, we followed a common standard recipe (62) where only the yeast strain was changed. Water (5 liters) was heated to a pasteurization temperature of 72°C. Malt extract was added to a final concentration of 100 g/liter, while thoroughly stirring, and allowed to infuse together for 30 min in temperatures between 63 and 67°C. The solution was then heated to 100°C, and once boiling had occurred, 1 g/liter of hops was added. The mixture was allowed to boil for 45 more minutes, followed by the addition of another 1 g/liter of hops. The mixture was then heated for an additional minute. Previously prepared ice-cold water was then added to the mixture, and the prepared wort was transferred to a sanitized fermentor and brought to a final volume of 10 liters. The wort was left at room temperature for 30 min before being divided into fermentation vessels and then overnight cultures of yeast were added. Fermentation typically began within 12 to 48 h, and the mixture was left untouched for a week.

### HS-SPME procedure and GC-MS analysis of beer.

The method we used was based on the method described by Rodriguez et al. (66). Beer bottles were cooled at 4°C to prevent loss of volatiles. The beer sample (6 ml), a magnetic stirrer, 100 μl of an internal standard (5 ppm 2-octanol) and 1.8 g of NaCl were added to 20-ml SPME headspace vials and were sealed with a polytetrafluoroethylene (PTFE)-silicon septum (Supelco). The samples were then incubated for 10 min at 44.8°C in a water bath on a heating plate and stirred by magnetic stirrer. The septum covering the vial headspace was pierced with the needle containing the SPME fiber and retracted, and the fiber was subsequently exposed to the headspace for 47 min at 44°C and then inserted directly into the GC-MS injection port. SPME fiber, consisting of 50/30-μm divinylbenzene/carboxen/polydimethylsiloxane (DVB/CAR/PDMS) with a length of 2 cm, and the manual holder were purchased from Supelco (Sigma-Aldrich). The analyses were performed using a gas chromatograph (Agilent 6890N) fitted with splitless injection with a liner suitable for SPME analysis and an Agilent 5973 mass spectrometer (MS) detector in full-scan mode. Agilent MSD ChemStation software was used to control the gas chromatograph (G1701-90057). Ultrahigh-purity-grade helium was used as the carrier gas at a flow rate of 1 ml/min. Samples were analyzed on a DB-5MS UI column (30 m by 0.250-mm inside diameter by 0.25-μm film thickness) from Agilent. The oven temperature was programmed as follows: 40°C as initial temperature, held for 5 min, followed by a ramp of temperature at 4°C/min to 60°C and then at 8°C/min to 200°C, then held for 15 min, holding at this temperature for 5 min. An electron impact ionization technique was used at 70 eV. The detector range of the scan was from *m*/*z* 10 to 250. Suggestions for the identification of the detected peaks were carried out by the Wiley mass spectrometry database. Peak areas were calculated using the integration order in the ChemStation software. For each sample, we determined the peak area for 2-octanol standard and ethanol, as well as for 35 aroma compounds usually found in beer. Following the integration of the 2-octanol peaks, we were not satisfied with its repeatability between technical repeats. Thus, we decided to use the peak areas of ethanol, which was separated clearly and was highly correlated to its determination by distillation in our lab, as an internal standard for each sample. To achieve normalized ethanol peak areas, we divided the peak area of ethanol by its concentration (percentage) determined by distillation, for each beer sample. Finally, the relative peak area for each compound was calculated by dividing the peak area of the compound to that of the normalized ethanol peak area, and multiplied by 1,000, to get more presentable numbers. This allows us a presentation of qualitative analysis of relative peak areas for each compound across our samples. The results presented and the statistical analysis were done by averaging the three biological samples for each yeast strain.

### Determination of carbohydrates in beers.

Stock solutions of phenol (J&K Scientific Gmbh) at 0.05 g/ml and d(+)-glucose (Merck) at 100 μg/ml were prepared. Glucose standards in aliquots of 1, 2, 3, 4, 5, 8, 10, 13, and 20 ml of the glucose stock solution were pipetted and transferred into nine 30-ml beakers. An adequate amount of distilled water was added to make a final volume of 20 ml. Each solution (2 ml) was measured and transferred into 10 test tubes. The phenol (2 ml) and 10 ml of the concentrated 95 to 97% sulfuric acid (Merck) were pipetted and added to each of the 10 test tubes. A light orange color developed, and the tube was allowed to stand for 10 min. The solutions were then transferred into 1-cm path-length cuvettes, and the absorbances were measured at 485 nm with a UV spectrophotometer (Genesys 10S UV Vis, Thermo). For measurements, 1 ml of beer was measured and transferred into a 1-liter volumetric flask. Distilled water was added to make a 1,000-ml solution. Aliquots (2 ml) were transferred into test tubes and mixed with 2 ml phenol solution and 10 ml concentrated sulfuric acid. A light orange color developed, and the absorbance was measured at 485 nm after 10 min. Results were determined by averaging triplicate measurements. The ethanol concentration in beer samples was determined using a Super Dee digital distillator and a Super Alcomat electronic hydrostatic balance (Gibertini, Italy). pH values of beer samples were measured using a Hanna HI 2211 pH meter (Hanna Instruments).

To analyze their spectrophotometric properties, the beer samples were degassed and centrifuged followed by a spectrophotometric (Genesys 10S UV Vis; Thermo Scientific) measurement at 430 nm (10-mm quartz cuvettes). Beer color was calculated by two scales: SRM and EBC, where SRM = absorbance × 12.7 and EBC = absorbance × 25.0. To determine the beers’ density, we used a hydrometer (“Alla” Franc).

### Beer tasting.

The flavor and aroma assessments were performed according to the BJCP’s judge procedure manual (https://www.bjcp.org/judgeprocman.php) as follows. A 100-ml sample was served to the assessors in identical vessels to prevent variations of aroma and flavor compound distribution. The assessors then recorded their impressions discreetly on a recognized form to avoid bias between the tasters. The forms, interdivided according to the subject’s appearance, aroma, flavor, and overall impression, were then collected, summarized, and processed. The summary ignored the appearance and overall impression sections, as well as hop flavor and aroma entries, and focused primarily on known fermentation by-products and sugar residue compounds. All “named entries” on the forms (such as caramel/fruity/etc.) come with a notation of the strength of the flavor/aroma derives from on a scale of 1 to 5 (left column on the evaluation form) and averaged by 5 testers.

### Statistical analysis.

Statistical analysis was performed using R (https://www.r-project.org) and Prism Graphpad 7 (https://www.graphpad.com/scientific-software/prism/). Differences between growth curves in wort medium (Fig. 3) were calculated using R “growthcurver” package (https://cran.r-project.org/web/packages/growthcurver/vignettes/Growthcurver-vignette.html) by fitting the growth data to the logistic equation. The *r* parameters of each curve were compared either to that of SafAle S.04, a modern beer yeast that served as a control, or to each other using principal-component analysis (PCA) with the R prcomp() command. For significance distances from the control growth curve, we used the Student's *t* test. Differences between aromatic and flavor compounds in beer produced by the isolated yeast strains (Fig. 4) and the aromas and flavors of these beer were compared by clustering analysis using R function hclust()with the method “complete” and the dist() function with the method “euclidean.” The dendrograms and clusters were created using Ward hierarchical clustering with bootstrapped *P* values using the R pvclust() method from R package pvclust, with parameters hclust="ward.D2” and method.dist="Euclidean.”

### Data availability.

Raw reads are available in GenBank under accession no. PRJNA449847. Genetic sequence analysis input and output files are available in the figshare repository (https://figshare.com), with accession numbers as provided in the figure legends.
