# Supplementary material for: Isolation and Characterization of Live Yeast Cells from Ancient Vessels as a Tool in Bio-Archaeology
Source: mBio. 2019 Apr 30;10(2):e00388-19. doi: 10.1128/mBio.00388-19 (PMC6495373; doi:10.1128/mBio.00388-19)
Supplement: TABLE S1 [file mBio.00388-19-st001.docx]

| **Index** | **Sample** | **Site** | **Sample Type** | **Isolated Yeast (+/-)** | **Beer production (+/-)** | **Isolated Yeast Name** |
| --- | --- | --- | --- | --- | --- | --- |
| **A. Putative beer vessels** | | |  |  |  |  |
| 1 | EB1 | En-Besor | Putative beer basin sherd | **+** | **+** | EBEgT12 |
| 2 | EB2 | En-Besor | Putative beer basin sherd | **+** | **+** | EBEgB8 |
| 3 | EB3 | En-Besor | Putative beer Holemouth sherd | **-** | **-** |  |
| 4 | TLV1 | Ha-Masger St. | Putative beer basin sherd | **+** | **+** | TLVEgRD4 |
| 5 | TLV2 | Ha-Masger St. | Putative beer basin sherd | **-** | **-** |  |
| 6 | RR1 | Ramat Rachel | Sherd from putative mead storage jar no. 9646\4 | **+** | **+** | RRPrTmd13 |
| 7 | RR2 | Ramat Rachel | Base of putative mead storage jar no. 9814/4 | **-** | **-** |  |
| 8 | RR3 | Ramat Rachel | Sherd from putative mead storage jar no. 9646/1 | **-** | **-** |  |
| 9 | RR4 | Ramat Rachel | Sherd from putative mead storage jar 9661/2 | **-** | **-** |  |
| 10 | TS1 | Tell es-Safi | Sediment in putative beer Vessel 1 | **-** | **-** |  |
| 11 | TS2 | Tell es-Safi | Sediment in putative beer Vessel 2 | **-** | **-** |  |
| 12 | TS3 | Tell es-Safi | Surface of large putative beer sherd 1 | **-** | **-** |  |
| 13 | TS4 | Tell es-Safi | Surface of large putative beer sherd 2 | **-** | **-** |  |
| 14 | TS5 | Tell es-Safi | Sediment from bottom of putative beer vessel | **-** | **-** |  |
| 15 | TS6 | Tell es-Safi | Sherd from bottom of putative beer vessel | **-** | **-** |  |
| 16 | TS7 | Tell es-Safi | Surface of bottom of putative beer vessel | **-** | **-** |  |
| 17 | TS8 | Tell es-Safi | Surface of upper parts of putative beer vessel | **-** | **-** |  |
| 18 | TS9 | Tell es-Safi | Surface of lower putative beer vessel 2 | **-** | **-** |  |
| 19 | TS10 | Tell es-Safi | Surface of large putative beer vessel | **+** | **+** | TZPlpvs7 |
| 20 | TS11 | Tell es-Safi | Putative beer Jug 2 | **+** | **+** | TZPlpvs2 |
| 21 | TS12 | Tell es-Safi | Putative beer Jug 2 | **-** | **-** |  |
| **B. Controls** |  |  |  |  |  |  |
| 22 | EB4 | En-Besor | Stone | **-** | **-** |  |
| 23 | EB5 | En-Besor | Stone | **-** | **-** |  |
| 24 | EB6 | En-Besor | Stone | **-** | **-** |  |
| 25 | EB7 | En-Besor | Stone | **-** | **-** |  |
| 26 | EB8 | En-Besor | Stone | **+** | **-** | EB8EgSt33 |
| 27 | EB9 | En-Besor | Stone | **-** | **-** |  |
| 28 | EB10 | En-Besor | Agricultural Sediment | **-** | **-** |  |
| 29 | EB11 | En-Besor | Agricultural Sediment | **-** | **-** |  |
| 30 | EB12 | En-Besor | Agricultural Sediment | **-** | **-** |  |
| 31 | EB13 | En-Besor | Agricultural Sediment | **-** | **-** |  |
| 32 | EB14 | En-Besor | Agricultural Sediment | **-** | **-** |  |
| 33 | Agr1 | Ma'on | Agricultural Sediment | **-** | **-** |  |
| 34 | Agr2 | Ma'on | Agricultural Sediment | **-** | **-** |  |
| 35 | Agr3 | Ma'on | Agricultural Sediment | **-** | **-** |  |
| 36 | Agr4 | Ma'on | Agricultural Sediment | **-** | **-** |  |
| 37 | Agr5 | Ma'on | Agricultural Sediment | **-** | **-** |  |
| 38 | Agr6 | Ma'on | Agricultural Sediment | **-** | **-** |  |
| 39 | Agr7 | Ma'on | Agricultural Sediment | **-** | **-** |  |
| 40 | Agr8 | Ma'on | Agricultural Sediment | **-** | **-** |  |
| 41 | Agr9 | Ma'on | Agricultural Sediment | **-** | **-** |  |
| 42 | Agr10 | Ma'on | Agricultural Sediment | **-** | **-** |  |
| 43 | Agr11 | Ma'on | Agricultural Sediment | **-** | **-** |  |
| 44 | Agr12 | Ma'on | Agricultural Sediment | **-** | **-** |  |
| 45 | Agr13 | Ma'on | Agricultural Sediment | **-** | **-** |  |
| 46 | Agr14 | Ma'on | Stone in Agricultural Sediment | **-** | **-** |  |
| 47 | Agr15 | Ma'on | Stone in Agricultural Sediment | **-** | **-** |  |
| 48 | Agr16 | Ma'on | Stone in Agricultural Sediment | **-** | **-** |  |
| 49 | Agr17 | Ma'on | Stone in Agricultural Sediment | **-** | **-** |  |
| 50 | Agr18 | Ma'on | Stone in Agricultural Sediment | **-** | **-** |  |
| 51 | Agr19 | Ma'on | Stone in Agricultural Sediment | **-** | **-** |  |
| 52 | Agr20 | Ma'on | Stone in Agricultural Sediment | **-** | **-** |  |
| 53 | Agr21 | Ma'on | Stone in Agricultural Sediment | **-** | **-** |  |
| 54 | Agr22 | Ma'on | Stone in Agricultural Sediment | **-** | **-** |  |
| 55 | Agr23 | Ma'on | Stone in Agricultural Sediment | **-** | **-** |  |
| 56 | Agr24 | Ma'on | Stone in Agricultural Sediment | **-** | **-** |  |
| 57 | Agr25 | Ma'on | Stone in Agricultural Sediment | **-** | **-** |  |
| 58 | Agr26 | Ma'on | Plant in Agricultural Sediment | **-** | **-** |  |
| 59 | Agr27 | Ma'on | Plant in Agricultural Sediment | **-** | **-** |  |
| 60 | RR5 | Ramat Rachel | Sediment from 9675 | **-** | **-** |  |
| 61 | RR6 | Ramat Rachel | Sherd from juglet 9618/6 | **-** | **-** |  |
| 62 | RR7 | Ramat Rachel | Sediment from 9592/1 | **-** | **-** |  |
| 63 | RR8 | Ramat Rachel | Sediment from 9675 | **-** | **-** |  |
| 64 | RR9 | Ramat Rachel | Sherd from storage jar 9618/6 | **-** | **-** |  |
| 65 | RR10 | Ramat Rachel | Sediment from 9592/1 | **-** | **-** |  |
| 66 | RR11 | Ramat Rachel | Sediment from Modern construction | **-** | **-** |  |
| 67 | RR12 | Ramat Rachel | Sediment from pit 12174 | **-** | **-** |  |
| 68 | RR13 | Ramat Rachel | Sediment from pit 12174 | **-** | **-** |  |
| 69 | RR14 | Ramat Rachel | Sediment from pit 12174 | **-** | **-** |  |
| 70 | RR15 | Ramat Rachel | Pit wall | **-** | **-** |  |
| 71 | RR16 | Ramat Rachel | Sediment from pit 12174 | **-** | **-** |  |
| 72 | RR17 | Ramat Rachel | Sediment from pit 12174 | **-** | **-** |  |
| 73 | RR18 | Ramat Rachel | Sediment from pit 12174 | **-** | **-** |  |
| 74 | RR19 | Ramat Rachel | Sediment from pit 12174 | **-** | **-** |  |
| 75 | RR20 | Ramat Rachel | Sediment from pit 12174 | **-** | **-** |  |
| 76 | RR21 | Ramat Rachel | Sediment from pit 12174 | **-** | **-** |  |
| 77 | RR22 | Ramat Rachel | Pit wall | **-** | **-** |  |
| 78 | RR23 | Ramat Rachel | Iron Age Wall | **-** | **-** |  |
| 79 | RR24 | Ramat Rachel | Sediment from pit 12174 | **-** | **-** |  |
| 80 | RR25 | Ramat Rachel | Sediment from pit 12174 | **-** | **-** |  |
| 81 | RR26 | Ramat Rachel | Sediment from pit 12174 | **-** | **-** |  |
| 82 | RR27 | Ramat Rachel | Sediment from pit 12174 | **-** | **-** |  |
| 83 | RR28 | Ramat Rachel | Sediment from pit 12174 | **-** | **-** |  |
| 84 | RR29 | Ramat Rachel | Sediment from pit 12174 | **-** | **-** |  |
| 85 | RR30 | Ramat Rachel | Sediment from pit 12174 | **-** | **-** |  |
| 86 | RR31 | Ramat Rachel | Sediment from pit 12174 | **-** | **-** |  |
| 87 | RR32 | Ramat Rachel | Sediment from pit 12174 | **-** | **-** |  |
| 88 | RR33 | Ramat Rachel | Sediment from pit 12174 | **-** | **-** |  |
| 89 | RR34 | Ramat Rachel | Sediment from pit 12174 | **-** | **-** |  |
| 90 | RR35 | Ramat Rachel | Sediment from pit 12174 | **-** | **-** |  |
| 91 | RR36 | Ramat Rachel | Sediment from pit 12174 | **-** | **-** |  |
| 92 | RR37 | Ramat Rachel | Sediment from pit 12174 | **-** | **-** |  |
| 93 | TS13 | Tell es-Safi | Holemouth jar 1 | **-** | **-** |  |
| 94 | TS14 | Tell es-Safi | Holemouth jar 2 | **-** | **-** |  |
| 95 | TS15 | Tell es-Safi | Holemouth jar surface | **-** | **-** |  |
| 96 | TS16 | Tell es-Safi | Small strainer 1 | **-** | **-** |  |
| 97 | TS17 | Tell es-Safi | Small strainer 2 | **-** | **-** |  |
| 98 | TS18 | Tell es-Safi | Holemouth jar 1 | **-** | **-** |  |
| 99 | TS19 | Tell es-Safi | Holemouth jar 2 | **-** | **-** |  |
| 100 | TS20 | Tell es-Safi | Holemouth jar surface | **-** | **-** |  |
| 101 | TS21 | Tell es-Safi | Small strainer 1 | **-** | **-** |  |
| 102 | TS22 | Tell es-Safi | Small strainer 2 | **-** | **-** |  |
| 103 | TS23 | Tell es-Safi | Sediment | **+** | **-** | TS23PlSt34 |
| 104 | TS24 | Tell es-Safi | Sediment | **-** | **-** |  |
| 105 | TS25 | Tell es-Safi | Sediment | **-** | **-** |  |
| 106 | TS26 | Tell es-Safi | Sediment | **-** | **-** |  |
| 107 | TS27 | Tell es-Safi | Sediment | **-** | **-** |  |
| 108 | TS28 | Tell es-Safi | Sediment | **-** | **-** |  |
| 109 | TS29 | Tell es-Safi | Sediment | **-** | **-** |  |
| 110 | TS30 | Tell es-Safi | Sediment | **-** | **-** |  |
| 111 | TS31 | Tell es-Safi | Sediment | **-** | **-** |  |
| 112 | TS32 | Tell es-Safi | Sediment | **-** | **-** |  |
| 113 | TS33 | Tell es-Safi | Sediment | **-** | **-** |  |
| 114 | TS34 | Tell es-Safi | Sediment | **-** | **-** |  |
| 115 | TS35 | Tell es-Safi | Sediment | **-** | **-** |  |
| 116 | TS36 | Tell es-Safi | Sediment | **-** | **-** |  |
| 117 | TS37 | Tell es-Safi | Sediment | **-** | **-** |  |
| 118 | TS38 | Tell es-Safi | Sediment | **-** | **-** |  |
| 119 | TS39 | Tell es-Safi | Sediment | **-** | **-** |  |
| 120 | TS40 | Tell es-Safi | Sediment | **-** | **-** |  |
| 121 | TS41 | Tell es-Safi | Sediment | **-** | **-** |  |
| 122 | TS42 | Tell es-Safi | Sediment | **-** | **-** |  |
| 123 | TS43 | Tell es-Safi | Sediment | **-** | **-** |  |
| 124 | TS44 | Tell es-Safi | Sediment | **-** | **-** |  |
| 125 | TS45 | Tell es-Safi | Sediment | **-** | **-** |  |
| 126 | TS46 | Tell es-Safi | Sediment | **-** | **-** |  |
| 127 | TS47 | Tell es-Safi | Sediment | **-** | **-** |  |
| 128 | TS48 | Tell es-Safi | Sediment | **-** | **-** |  |
| 129 | TS49 | Tell es-Safi | Sediment | **-** | **-** |  |
| 130 | TS50 | Tell es-Safi | Sediment | **-** | **-** |  |
| 131 | TS51 | Tell es-Safi | Sediment | **-** | **-** |  |
| 132 | TS52 | Tell es-Safi | Sediment | **-** | **-** |  |
| 133 | TS53 | Tell es-Safi | Sediment | **-** | **-** |  |
| 134 | TS54 | Tell es-Safi | Sediment | **-** | **-** |  |
| **C. Lamps** |  |  |  |  |  |  |
| 135 | RR38 | Ramat-Rachel | Lamp 1 | **+** | **-** | RRPrNerP7 |
| 136 | RR39 | Ramat-Rachel | Lamp 2 | **-** | **-** |  |
| 137 | RR40 | Ramat-Rachel | Lamp 3 | **-** | **-** |  |
| 138 | TS55 | Tell es-Safi | Lamp 1 | **+** | **-** | TS55Pllmp35 |
| 139 | TS56 | Tell es-Safi | Lamp 2 | **+** | **-** | TS55Pllmp36 |
| 140 | TS57 | Tell es-Safi | Lamp 3 | **-** | **-** |  |
