# Supplementary material for: Isolation and Characterization of Live Yeast Cells from Ancient Vessels as a Tool in Bio-Archaeology
Source: mBio. 2019 Apr 30;10(2):e00388-19. doi: 10.1128/mBio.00388-19 (PMC6495373; doi:10.1128/mBio.00388-19)
Supplement: TABLE S2 [file mBio.00388-19-st002.docx]

|  | **Yeast** | **Site** | **Source** | **ITS Identification** | **Score** | **Expect** | **Identities** | **Gaps** | **Drinkable beer production** |  |  |
| --- | --- | --- | --- | --- | --- | --- | --- | --- | --- | --- | --- |
|  | **Yeast from putative beer vessels** | | | | | | | | | |  |
| 1 | EBEgT12 | En-Besor | Putative beer basin sherd | *Nakaseomyces delphensis* | 183 bits(99) | 2e-42 | 182/220(83%) | 20/220(9%) | + |  |  |
| **ITS Sequence:** AGTCCNGNTACNTTGNTGAAGTTTTAAAGTTATTTTTTCTTCACAAAAAGNAAAAATTAGTTTTGACATATTAAAATAAAAATAGTTGTTTGTGTTATTTCAACTTCTCCCCAGAAAGAGAAGAAGTAAGAAAAAAAGTACAAAAAGTAAAATACTCCAGTGTGTGTAAAATGATGTGGAACAAAAAGCATTCGCCCCCCGCTGCTGCCGCGCACTTAAGCGCAGGCCGCAAAACAGAAAACAAACATAATCGCTCTCGCTCCCCAGTCAATTTTTTTTTCTTTAATGATCCTTCCGCAGGTTCACCTACGGAAACCTTGTTACGACTTTTACTTCCTCTAAATGACCAAGAA | | | | | | | | | | | |
| 2 | EBEgB8 | En-Besor | Putative beer basin sherd | *Nakaseomyces delphensis* | 207 bits(112) | 1e-49 | 288/367(78%) | 39/367(10%) | **+/-** |  |  |
| **ITS Sequence:** CATCNANGGTNTCCGCAGTGAACCTGCGGAGGATNTTAAAGAAAAAAAAATTGACTGGGGAGCGAGAGCGATTATGTTTGTTTTCTGTTTTGCGGCCTGCGCTTAAGTGCGCGGCAGCAGCGGGGGGCGAATGCTTTTTGTTCCACATCATTTTACACACACTGGAGTATTTTACTTTTTGTACTTTTTTTCTTACTTCTTCTCTTTCTGGGGAGAAGTTGAAATAACACAAACAACTATTTTTATTTTAATATGTCAAAACTAATTTTTCTTTTTGTTGAAGAAAAAATAACTTTAAAACTTTCAACAATGGATCTCTTGGTTCTCGCATCGATGAAGAACNGCANCA | | | | | | | | | | | |
| 3 | TLVEgRD4 | Ha-Masger St. | Putative beer basin sherd | *Rhodotorula kratochvilovae* | 401 bits(217) | 4e-108 | 220/221(99%) | 1/221(0%) | **-** |  |  |
| **ITS Sequence:** CNGNNACGCGCNGTGAACCTGCGGAGGAtcaTTAGTGAATCTAGGGTGTCCAATTTAACTTGGAGCCCGAACTCTCACTTTCTAACCCTGTGCATCTGTTATTGGTTAGTAGCTCTTCGGAGTGAACTCCATTCACTTACAAACACAAAGTCTATGAATGTATACAAAATTATAACAAAACAAAACTTTCAACAACGGATCTCTTGGCTCTCGCATCGATGAAGAACGCAGCA | | | | | | | | | | | |
| 4 | TZPlpvs7 | Tell es-Safi | Surface of large putative beer vessel | *Schwanniomyces occidentalis/ Debaryomyces castellii* | 464 bits(251) | *6e-127* | *272/282(96%)* | *2/282(0%)* | **+/-** |  |  |
| **ITS Sequence:** TACAGGTTTCCCGTCGGTGAACCTGCGGaAGGAtcaTTACAGTATTCCTTTTTGCCaGCGCTTAATTGCGCGGCGAAAAAACCTTACACACAGTGTTTTTTGTTATTACAAGAACTTTTGCTTTGGTCTGTCTCTAGAAATAGAGTTGGGCCAGAGGTTTAACTAAACTTCAATTTTATATTGAATTGTTTTTTAATTAATTGTCAATTTGTTGATTAAATTCAAAAAATCTTCAAAACTTTCAACAACGGATCTCTTGGTTCTCGCATCGATGAAGAACGCAGCA | | | | | | | | | | | |
| 5 | TZPlpvs2 | Tell es-Safi | Putative beer Jug 2 | *Saccharomyces cerevisiae* | 787 bits(426) | 0.0 | 428/430(99%) | 0/430(0%) | **+** |  |  |
| **ITS Sequence:** GAACCTGCGGaAGGATcaTTAAAGAAATTTAATAATTTTGAAAATGGATTTTTTTGTTTTGGCAAGAGCATGAGAGCTTTTACTGGGCAAGAAGACAAGAGATGGAGAGTCCAGCCGGGCCTGCGCTTAAGTGCGCGGTCTTGCTAGGCTTGTAAGTTTCTTTCTTGCTATTCCAAACGGTGAGAGATTTCTGTGCTTTTGTTATAGGACAATTAAAACCGTTTCAATACAACACACTGTGGAGTTTTCATATCTTTGCAACTTTTTCTTTGGGCATTCGAGCAATCGGGGCCCAGAGGTAACAAACACANACAATTTTATCTATTCATTAAATTTTTGTCAAAAACAAGAATTTTCGTAACTGGNAATTTTAAAATATTAAAAACTTTCAACAACGGATCTCTTGGTTCTCGCATCGATGAAGAACGCANCACA | | | | | | | | | | | |
| 6 | RRPrTmd13 | Ramat Rachel | Sherd from putative mead storage jar no. 9646\4 | *Hyphopichia burtonii* | 189 bits(102) | 2e-44 | 138/154(90%) | 7/154(4%) | **+** |  |  |
| **ITS Sequence:** GANCGAGTTTTGATTTTTGTATTGGTTGACTTTATATAAGTTGGTTAGTTGTAGTATAAACTTAATCGTGTAAACAAATAAATTATTAATGATCCTTCCGCAGGTTCACCTACGGAAACCTTGTTACGACTTTTACTTCCTCTAAATGACCAAGA | | | | | | | | | | | |
|  | **Yeast from Lamps** | | | | | | | | | |  |
| 7 | RRPrNerP7 | Ramat-Rachel | Lamp 1 | *Hyphopichia burtonii* | 187 bits(101) | 7e-44 | 135/150(90%) | 8/150(5%) | **-** |  |  |
| **ITS Sequence:**  GAAGTTTTGATTTTTGTATTGGTTGACTTTATATAAGTTGGTTAGTTGTAGTATAAACTTAATCGTGTAAACAAATAAATTATTAATGATCCTTCCGCAGGTTCACCTACGGAAACCTTGTTACGACTTTTACTTCCTCTAAATGACCAAGA | | | | | | | | | | | |
| 8 | TS55Pllmp35 | Tell es-Safi | Lamp 1 | *Yarrowia lipolytica* | 244 bits(132) | 4e-61 | 141/146(97%) | 2/146(1%) | **-** |  |  |
| **ITS Sequence:** TCTAAGGTTTCCGNAGTGAACCTGCGGAAGGANATTATTGATTTTATCTATTTCTGTGGATTTCTATTCTATTACAGCGTCATTTTATCTCAATTATAACTATCAACAACGGATCTCTTGGCTCTCGCATCGATGAAGAACGCAGCAC | | | | | | | | | | | |
| 9 | TS55Pllmp36 | Tell es-Safi | Lamp 2 | *Yarrowia lipolytica* | 246 bits(133) | 1e-61 | 141/146(97%) | 1/146(0%) | **-** |  |  |
| **ITS Sequence:**  ATCTANGGTTTCCGTNGGTGAACCTGCGGAGGATCNTTATTGATTTTATCTATTTCTGTGGATTTCTATTCTATTACAGCGTCATTTTATCTCAATTATAACTATCAACAACGGATCTCTTGGCTCTCGCATCGATGAAGAACGCAGCA | | | | | | | | | | | |
|  | **Yeast from Controls** | | | | | | | | | |  |
| 10 | EB8EgSt33 | En-Besor | Stone | *Candida albicans* | 403 bits(218) | 1e-108 | 221/222(99%) | 1/222(0%) | **-** |  |  |
| **ITS Sequence:**  TNTACGGTTTCCGTAGGTGAACCTGCGGaAGGATcTTACTGATTTGCTTAATTGCACCACATGTGTTTTTCTTTGAAACAAACTTGCTTTGGCGGTGGGCCCAGCCTGCCGCCAGAGGTCTAAACTTACAACCAATTTTTTATCAACTTGTCACACCAGATTATTACTAATAGTCAAAACTTTCAACAACGGATCTCTTGGTTCTCGCATCGATGAAGAACGCAGCA | | | | | | | | | | | |
| 11 | TS23PlSt34 | Tell es-Safi | Sediment | Unidentified | NA | NA | NA | NA | **-** |  |  |
| **ITS Sequence:**  TCTACGGTTTCCGTGGTGAACCTGCGGNAGGATNTTAATAAACGAATTCTTTTTGTGAATTNCTNANCTTTTTNANNCGANTCANTCTTTNAATTATNACTCTCNACNGNGGNTCTCGGGGCTCNCGCNNCGGNGAAGAGCGCC | | | | | | | | | | | |
