# Supplementary figures and images for: Isolation and Characterization of Live Yeast Cells from Ancient Vessels as a Tool in Bio-Archaeology
Source: mBio. 2019 Apr 30;10(2):e00388-19. doi: 10.1128/mBio.00388-19 (PMC6495373; doi:10.1128/mBio.00388-19)

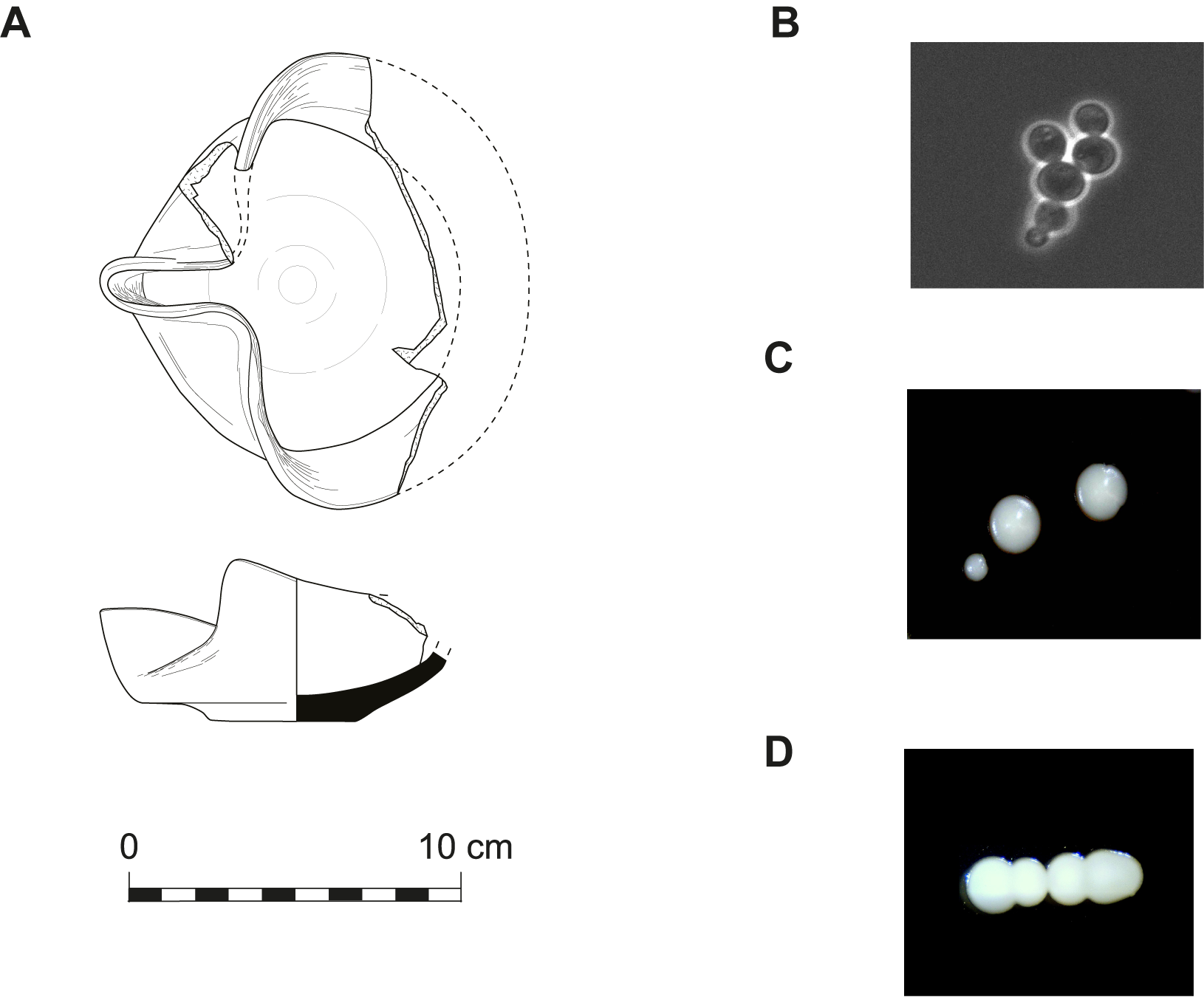

Supplement: FIG S1 [file mBio.00388-19-sf001.tif]

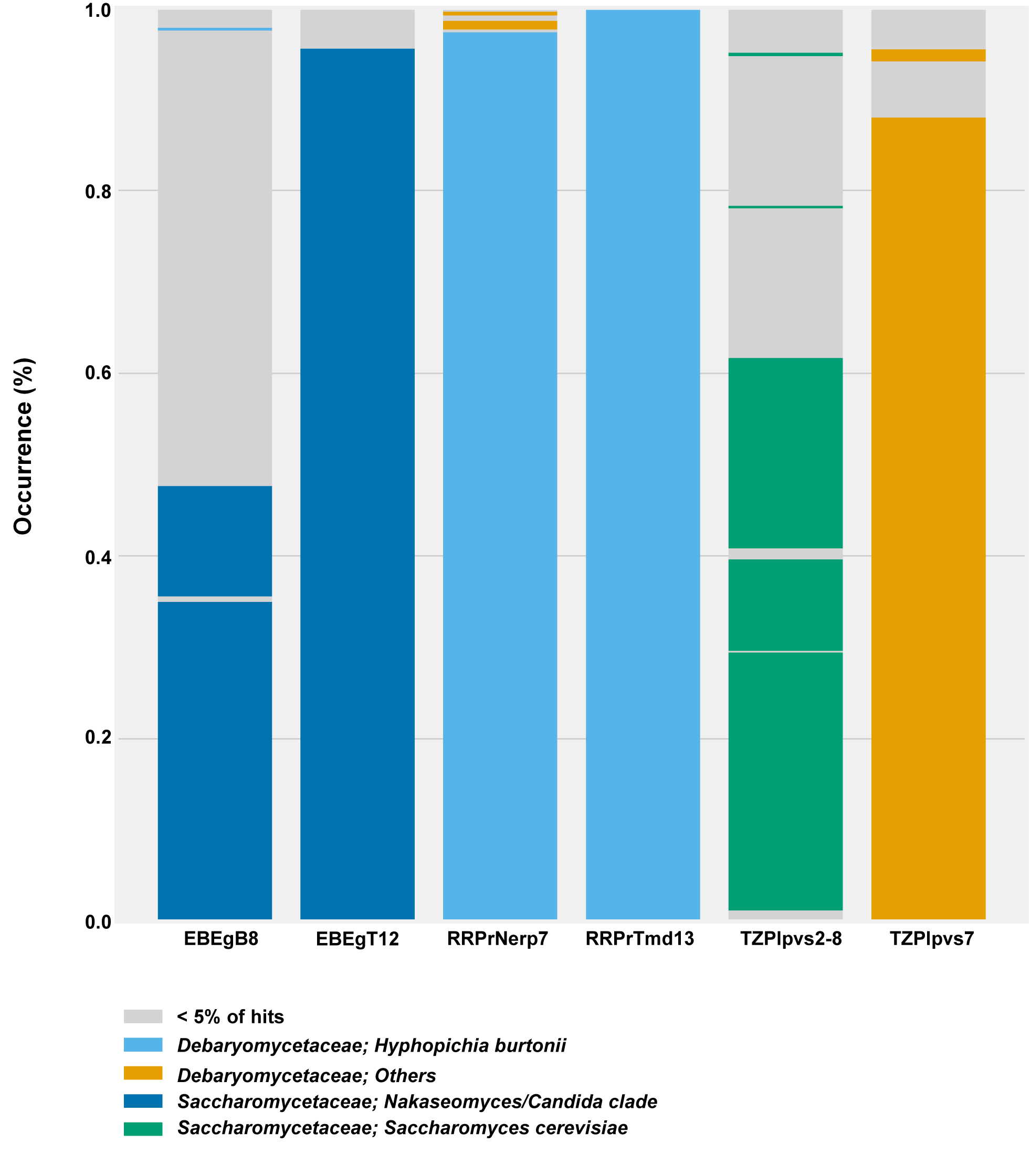

Supplement: FIG S2 [file mBio.00388-19-sf002.tif]

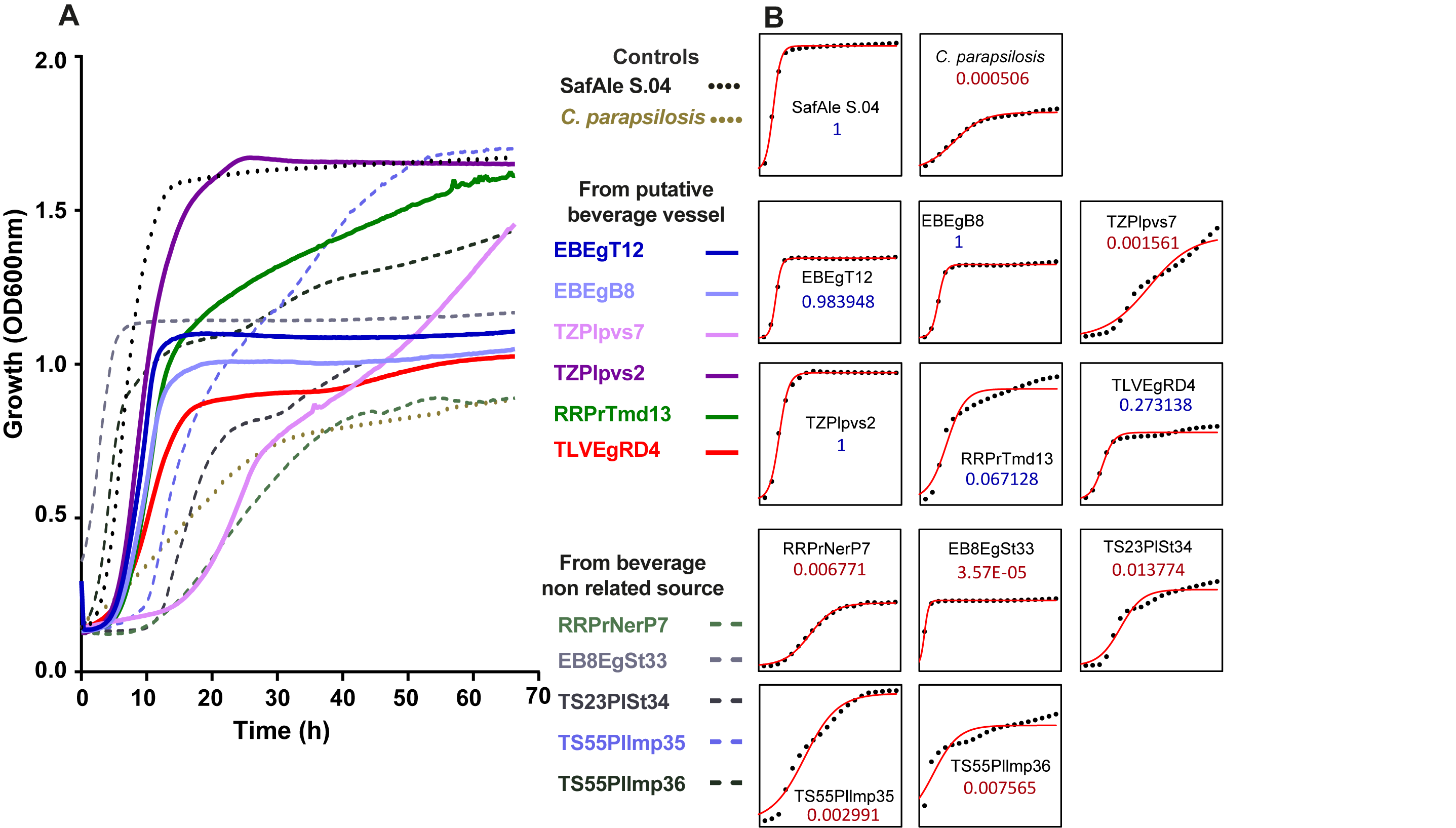

Supplement: FIG S3 [file mBio.00388-19-sf003.tif]

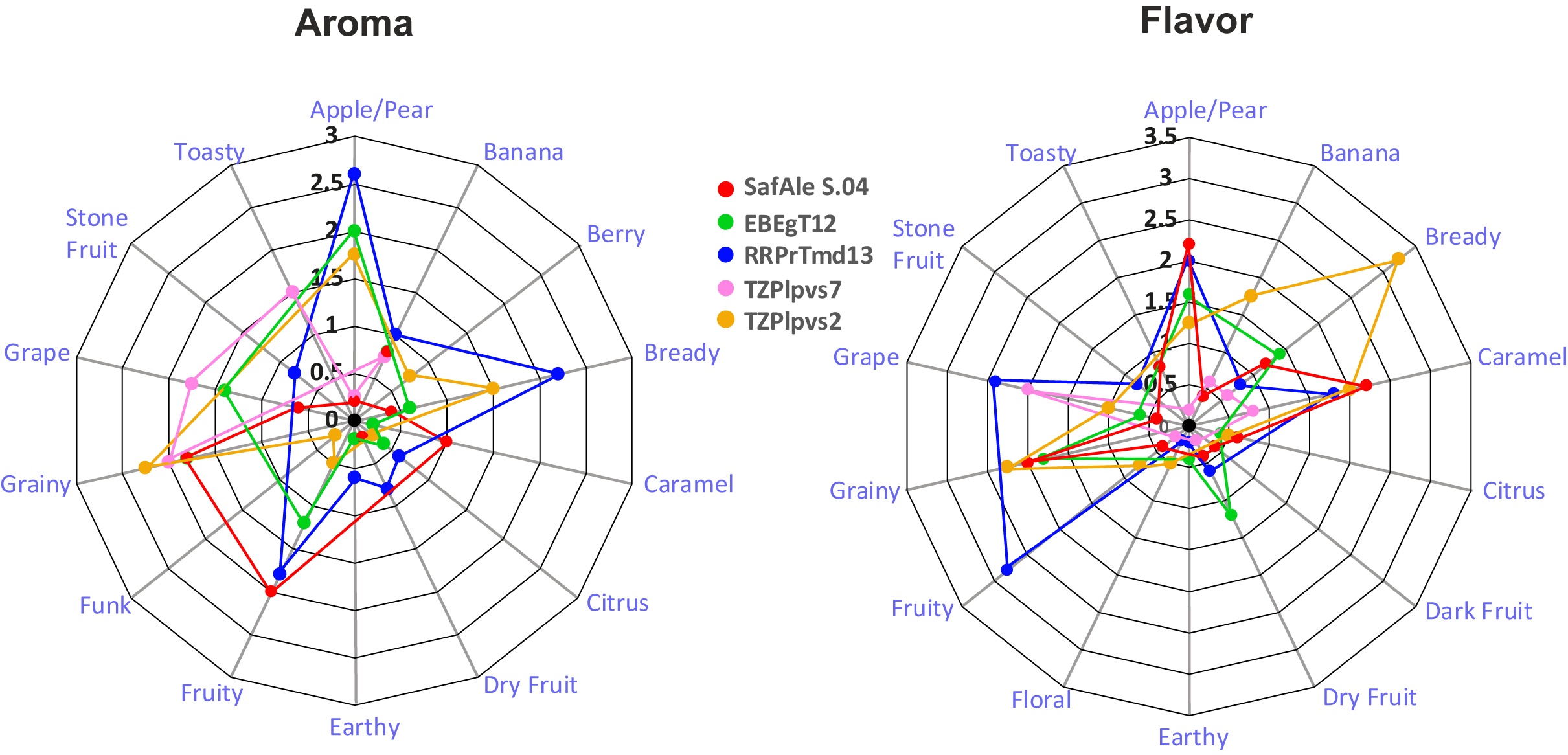

Supplement: FIG S4 [file mBio.00388-19-sf004.tif]
