# Supplementary material for: Isolation and Characterization of Live Yeast Cells from Ancient Vessels as a Tool in Bio-Archaeology
Source: mBio. 2019 Apr 30;10(2):e00388-19. doi: 10.1128/mBio.00388-19 (PMC6495373; doi:10.1128/mBio.00388-19)
Supplement: TABLE S4 [file mBio.00388-19-st004.docx]

| **GO term** | **Description** | **eggNOG ortholog** | **RRPrTmd13** | **RRPrNerP7** | **Type** |
| --- | --- | --- | --- | --- | --- |
| GO:0008643 | Carbohydrate transport | 03QDN | 1 | 0 | Addition in wine yeasts |
| GO:0015688 | Iron chelate transport | 04DYP | 3 | 1 |  |
| GO:0015891 | Siderophore transport | 04DYP | 3 | 1 |  |
| GO:0034219 | Carbohydrate transmembrane transport | 03QDN | 1 | 0 |  |
| GO:0035725 | Sodium ion transmembrane transport | 03JK3 | 2 | 1 |  |
|  | Sodium ion transmembrane transport | 03KFD | 1 | 0 |  |
| GO:0043328 | Protein targeting to vacuole involved in ubiquitin-dependent protein catabolic process via the multivesicular body sorting pathway | 03KWQ | 1 | 0 |  |
| GO:0055085 | Transmembrane transport | 03JK3 | 2 | 1 |  |
|  |  | 03IFM | 5 | 4 |  |
|  |  | 03K32 | 2 | 1 |  |
|  |  | 03K4P | 2 | 1 |  |
|  |  | 03JBP | 2 | 1 |  |
|  |  | 03K6X | 3 | 2 |  |
|  |  | 04DYP | 3 | 1 |  |
|  |  | 03MZC | 1 | 0 |  |
|  |  | 03PEK | 1 | 0 |  |
|  |  | 03JH4 | 1 | 0 |  |
|  |  | 03JHG | 1 | 0 |  |
|  |  | 03KFD | 1 | 0 |  |
|  |  | 03QDN | 1 | 0 |  |
|  |  | 04DHH | 1 | 0 |  |
|  |  | 03M3F | 1 | 0 |  |
|  |  | 03KX1 | 1 | 0 |  |
|  |  | 04DJ7 | 1 | 0 |  |
|  |  | 03K20 | 1 | 0 |  |
|  |  | 03IWW | 1 | 0 |  |
|  |  | 03SKF | 1 | 0 |  |
|  |  | 03JHH | 1 | 0 |  |
|  |  | 03Q2N | 1 | 0 |  |
|  |  | 03SRU | 1 | 0 |  |
|  |  | 03V0X | 1 | 0 |  |
| GO:1901678 | Iron coordination entity transport | 04DYP | 3 | 1 |  |
| GO:0005984 | Disaccharide metabolic process | 03KTY | 0 | 1 | Deletion in wine yeasts |
| GO:0009311 | Oligosaccharide metabolic process | 03KTY | 0 | 1 |  |
|  |  | 03MN8 | 0 | 1 |  |
| GO:0009313 | Oligosaccharide catabolic process | 03KTY | 0 | 1 |  |
| GO:0046352 | Disaccharide catabolic process | 03KTY | 0 | 1 |  |
