# Supplementary material for: Isolation and Characterization of Live Yeast Cells from Ancient Vessels as a Tool in Bio-Archaeology
Source: mBio. 2019 Apr 30;10(2):e00388-19. doi: 10.1128/mBio.00388-19 (PMC6495373; doi:10.1128/mBio.00388-19)
Supplement: TABLE S7 [file mBio.00388-19-st007.docx]

**Table S7**. Uploaded additional files 1-11

Genetic sequence analysis input and output files were uploaded and are available on FigShare (<https://figshare.com>), with DOI numbers as provided in the following legends.

**1) Uploaded file 1: Sequence alignment for figure 3a (118 orthologs)**DOI:10.6084/m9.figshare.6085952
A sequence alignment for the tree of 118 orthologs presented as Figure 3A. 
**2) Uploaded file 2: Partition file for figure 3a (118 orthologs)**
DOI:10.6084/m9.figshare.6086087
Partition file for the tree of 118 orthologs presented as Figure 3A.
**3) Uploaded file 3: Tree file for figure 3a (118 orthologs)**DOI:10.6084/m9.figshare.6086096
A sequence alignment for the tree of 118 orthologs presented as Figure 3A.
**4) Uploaded file 4: Sequence alignment for figure 3b (465 orthologs)**DOI:10.6084/m9.figshare.6086102
A sequence alignment for the tree of 465 orthologs presented as Figure 3B.
**5) Uploaded file 5: partition file for figure 3b (465 orthologs)**DOI:10.6084/m9.figshare.6086105
A partition file for the tree of 465 orthologs presented as Figure 3B.
**6) Uploaded file 6: Tree file for figure 3b (465 orthologs)**
DOI:10.6084/m9.figshare.6086114
A tree file for the tree of 465 orthologs presented as Figure 3B.
**7) Uploaded file 7: Sequence data for Ascomycota LSU-*rRNA* barcoding analysis**DOI:10.6084/m9.figshare.6086120
LSU-*rRNA* based molecular barcoding of yeasts, sequence data file.
**8) Uploaded file 8: Centroid sequences for Ascomycota LSU-*rRNA* barcoding analysis**DOI:10.6084/m9.figshare.6086126
LSU-*rRNA* based molecular barcoding of yeasts, centroid sequences file.
**9) Uploaded file 9: Aligned centroid sequences for Ascomycota LSU-*rRNA* barcoding analysis**DOI:10.6084/m9.figshare.6086129
LSU-*rRNA* based molecular barcoding of yeasts, centroid sequence alignment file.
**10) Uploaded file 10: Trimmed alignment of centroid sequences for Ascomycota LSU-*rRNA* barcoding** **analysis**
DOI:10.6084/m9.figshare.6086132
LSU-*rRNA* based molecular barcoding of yeasts, trimmed centroid sequence alignment file.
**11) Uploaded file 11: Tree file for Ascomycota LSU-*rRNA* barcoding analysis**DOI:10.6084/m9.figshare.6086138
LSU-*rRNA* based molecular barcoding of yeasts, phylogenetic tree file.
