# Supplementary material for: Isolation and Characterization of Live Yeast Cells from Ancient Vessels as a Tool in Bio-Archaeology
Source: mBio. 2019 Apr 30;10(2):e00388-19. doi: 10.1128/mBio.00388-19 (PMC6495373; doi:10.1128/mBio.00388-19)
Supplement: TABLE S6 [file mBio.00388-19-st006.docx]

**Table S6**

Qualitative analysis of aromatic and flavor compounds in beers produced by yeast strains from ancient vessels. qualitative analysis of relative peak areas for each compound across our samples was conducted using SPME Fiber (DVB/CAR/PDMS), followed by separation by gas chromatographer and detection by Agilent 5973 mass spectrometer (MS) detector in Full-Scan mode. Suggestions for the identification of the detected peaks were carried out by Wiley mass spectrometry database. Semi-quantitative analysis was based on the relative peak areas calculated using the integration order in the ChemStation Software and using the peak area of ethanol as an internal standard. Ethanol quantities in each sample were determined by distillation, and each peak was normalized in accord with the relevant ethanol quantity in each sample. The relative peak area for each compound was calculated by dividing the peak area of the compound to that of the normalized ethanol peak area, and multiplied in 1000, to get more presentable numbers.

| **Compounds** | **SafAle S.04** | **EBEgT12** | **TZPlpvs7** | **TZPlpvs2** | **RRPrTmd13** | **TLVEgRD4** |
| --- | --- | --- | --- | --- | --- | --- |
| **Isoamyl alcohol** | 937.62 | 1000.93 | 558.17 | 1343.13 | 1008.02 | 1069.36 |
| **Isoamyl acetate** | 517.23 | 783.36 | 169.3933 | 672.31 | 219.26 | 754.97 |
| **Styrene (Cinnamene )** | 0.00 | 37.69 | 98.57 | 0.00 | 9.55 | 0.00 |
| **Methionol** | 0.00 | 0.00 | 0.00 | 1.84 | 3.84 | 0.00 |
| **2-Octanone** | 3.31 | 2.51 | 0.00 | 53.35 | 0.00 | 3.58 |
| **Ethyl hexanoate** | 316.81 | 286.66 | 28.31 | 358.45 | 134.33 | 205.02 |
| **2-Ethyl-1-hexanol** | 29.32 | 40.66 | 63.83 | 7.91 | 50.36 | 23.44 |
| **Limonene** | 0.00 | 0.00 | 0.00 | 3.74 | 0.00 | 7.31 |
| **1-Octanol** | 16.99 | 14.97 | 15.86 | 17.87 | 11.96 | 11.13 |
| **2-Nonanone** | 8.12 | 2.25 | 0.00 | 3.82 | 0.00 | 2.16 |
| **Ethyl heptanoate** | 0.00 | 1.22 | 0.00 | 1.27 | 3.33 | 0.00 |
| **Linalool** | 40.81 | 59.81 | 78.60 | 96.49 | 62.39 | 34.23 |
| **Nonanal** | 8.43 | 0 | 8.68 | 0 | 6.64 | 10.23 |
| **Phenethyl alcohol** | 905.82 | 733.44 | 192.07 | 784.48 | 493.38 | 893.37 |
| **octyl acetate** | 4.29 | 10.58 | 8.20 | 0 | 1.43 | 6.17 |
| **1-Nonanol** | 6.00 | 0 | 6.41 | 0 | 2.18 | 0 |
| **Ethyl benzoate** | 4.32 | 5.92 | 0 | 9.03 | 3.86 | 6.76 |
| **Octanoic acid** | 20.01 | 12.61 | 0 | 114.23 | 0 | 10.88 |
| **Ethyl octanoate** | 962.16 | 917.24 | 252.04 | 1362.31 | 283.70 | 822.28 |
| **Decanal** | 1.89 | 0.89 | 1.75 | 9.67 | 2.56 | 4.29 |
| **Citronellol** | 6.73667 | 5.43 | 15.40667 | 101.77667 | 22.02667 | 4.76333 |
| **Geraniol** | 0 | 0 | 3.206667 | 8.3366667 | 2.22 | 0 |
| **Phenethyl acetate** | 383.91 | 632.66 | 46.06 | 445.08 | 300.32 | 405.60 |
| **1-Decanol** | 11.64 | 8.99 | 5.18 | 18.56 | 3.84 | 8.74 |
| **2-Undecanone** | 4.63 | 0 | 0 | 8.40 | 0.95 | 1.12 |
| **Ethyl nonanoate** | 5.24 | 9.87 | 4.00 | 9.14 | 1.03 | 3.17 |
| **2-Undecanol** | 2.16 | 2.31 | 0 | 0 | 3.30 | 0 |
| **Citronellol acetate** | 1.69 | 2.37 | 0.00 | 14.54 | 0.54 | 2.06 |
| **Ethyl 9-decenoate** | 66.19 | 39.71 | 19.70 | 284.31 | 23.02 | 38.08 |
| **Ethyl decanoate** | 423.70 | 589.59 | 46.92 | 1047.50 | 84.21 | 359.74 |
| **Decyl acetate** | 1.57 | 4.29 | 0 | 0 | 0 | 1.07 |
| **Ethyl laurate** | 46.78 | 95.31 | 5.49 | 240.21 | 14.30 | 35.10 |
| **Ethyl linoleate** | 0 | 0 | 0.31 | 0 | 0 | 0 |
| **2-Pentadecanone** | 0.85 | 3.55 | 0.92 | 0 | 0 | 0 |
| **2-Tridecanone** | 9.21 | 1.96 | 13.61 | 0 | 7.51 | 11.45 |
| **2-Pentadecanone_b** | 104.02 | 16.14 | 0 | 0 | 23.48 | 12.25 |
