# Supplementary material for: Isolation and Characterization of Live Yeast Cells from Ancient Vessels as a Tool in Bio-Archaeology
Source: mBio. 2019 Apr 30;10(2):e00388-19. doi: 10.1128/mBio.00388-19 (PMC6495373; doi:10.1128/mBio.00388-19)
Supplement: TABLE S3 [file mBio.00388-19-st003.docx]

| **GO term** | **Description** | **eggNOG ortholog** | **EBEgT12** | **EBEgB8** | **Type** |
| --- | --- | --- | --- | --- | --- |
| GO:0005984 | Disaccharide metabolic process | 03IFT | 2 | 1 | Addition in beer yeasts |
|  |  | 03KJX | 2 | 1 |  |
|  |  | 03KW1 | 2 | 1 |  |
| GO:0006081 | Cellular aldehyde metabolic process | 03JQS | 1 | 0 |  |
|  |  | 03JKG | 1 | 0 |  |
|  |  | 03IG0 | 1 | 0 |  |
| GO:0009110 | Vitamin biosynthetic process | 03IHX | 1 | 0 |  |
|  |  | 03KYR | 1 | 0 |  |
|  |  | 04E87 | 1 | 0 |  |
| GO:0042364 | Water-soluble vitamin biosynthetic process | 03IHX | 1 | 0 |  |
|  | Water-soluble vitamin biosynthetic process | 03KYR | 1 | 0 |  |
|  | Water-soluble vitamin biosynthetic process | 04E87 | 1 | 0 |  |
| GO:0042822 | Pyridoxal phosphate metabolic process | 03PA2 | 2 | 1 |  |
| GO:0042823 | Pyridoxal phosphate biosynthetic process | 03PA2 | 2 | 1 |  |
| GO:0043328 | Protein targeting to vacuole involved in ubiquitin-dependent protein catabolic process via the multivesicular body sorting pathway | 03K0Q | 2 | 1 |  |
|  |  | 03IVB | 2 | 1 |  |
|  |  | 03NY3 | 1 | 0 |  |
| GO:0008643 | Carbohydrate transport | 03N1H | 0 | 1 | Deletion in beer yeasts |
|  |  | 03J8X | 3 | 4 |  |
|  |  | 03RVN | 0 | 1 |  |
|  |  | 03MB9 | 0 | 1 |  |
| GO:0008645 | Hexose transport | 03N1H | 0 | 1 |  |
|  |  | 03J8X | 3 | 4 |  |
|  |  | 03RVN | 0 | 1 |  |
|  |  | 03MB9 | 0 | 1 |  |
| GO:0015749 | Monosaccharide transport | 03N1H | 0 | 1 |  |
|  |  | 03J8X | 3 | 4 |  |
|  |  | 03RVN | 0 | 1 |  |
|  |  | 03MB9 | 0 | 1 |  |
| GO:0015758 | Glucose transport | 03N1H | 0 | 1 |  |
|  |  | 03J8X | 3 | 4 |  |
|  |  | 03RVN | 0 | 1 |  |
| GO:0034219 | Carbohydrate transmembrane transport | 03MB9 | 0 | 1 |  |
| GO:0035428 | Hexose transmembrane transport | 03MB9 | 0 | 1 |  |
| GO:0035725 | Sodium ion transmembrane transport | 03JK3 | 0 | 2 |  |
| GO:0043328 | Protein targeting to vacuole involved in ubiquitin-dependent protein catabolic process via the multivesicular body sorting pathway | 03SGF | 0 | 1 |  |
|  |  | 03KAZ | 0 | 1 |  |
| GO:0044275 | Cellular carbohydrate catabolic process | 04E8B | 2 | 3 |  |
|  |  | 03KTY | 1 | 2 |  |
|  |  | 03I86 | 1 | 2 |  |
| GO:0055085 | Transmembrane transport | 03JK3 | 0 | 2 |  |
|  |  | 03I8D | 0 | 1 |  |
|  |  | 03K7D | 0 | 1 |  |
|  |  | 03IHF | 0 | 1 |  |
|  |  | 03KEF | 0 | 1 |  |
|  |  | 03IVI | 1 | 2 |  |
|  |  | 03M1M | 0 | 1 |  |
|  |  | 03K4P | 1 | 2 |  |
|  |  | 03IS9 | 0 | 2 |  |
|  |  | 04DIT | 1 | 3 |  |
|  |  | 03KUX | 0 | 1 |  |
|  |  | 03K6X | 0 | 1 |  |
|  |  | 03JW6 | 1 | 2 |  |
|  |  | 03J2Y | 1 | 2 |  |
|  |  | 03IE8 | 2 | 3 |  |
|  |  | 03RPI | 0 | 1 |  |
|  |  | 03PUA | 0 | 1 |  |
|  |  | 04DWX | 0 | 1 |  |
|  |  | 03M2I | 0 | 1 |  |
|  |  | 03JM4 | 0 | 1 |  |
|  |  | 03MQQ | 2 | 3 |  |
|  |  | 03TTF | 0 | 1 |  |
|  |  | 03IQY | 0 | 1 |  |
|  |  | 03RME | 0 | 1 |  |
|  |  | 03N4A | 1 | 2 |  |
|  |  | 03KF7 | 0 | 1 |  |
|  |  | 03URA | 0 | 1 |  |
|  |  | 03TA9 | 0 | 1 |  |
|  |  | 03I92 | 0 | 1 |  |
|  |  | 03K20 | 0 | 2 |  |
|  |  | 03R0H | 0 | 1 |  |
|  |  | 03IWW | 2 | 3 |  |
|  |  | 03SZR | 0 | 1 |  |
|  |  | 03IP6 | 0 | 2 |  |
|  |  | 03T0Y | 0 | 1 |  |
|  |  | 03ICG | 0 | 1 |  |
|  |  | 03IIH | 0 | 1 |  |
|  |  | 03J8X | 3 | 4 |  |
|  |  | 03IHP | 0 | 1 |  |
|  |  | 03IRD | 0 | 1 |  |
|  |  | 03MB9 | 0 | 1 |  |
|  |  | 03P9J | 0 | 1 |  |
|  |  | 03W01 | 0 | 1 |  |
|  |  | 03N8M | 0 | 1 |  |
|  |  | 03KCM | 0 | 1 |  |
|  |  | 03PG9 | 0 | 1 |  |
| GO:1901678 | Iron coordination entity transport | 03RZW | 0 | 1 |  |
