# Supplementary material for: Isolation and Characterization of Live Yeast Cells from Ancient Vessels as a Tool in Bio-Archaeology
Source: mBio. 2019 Apr 30;10(2):e00388-19. doi: 10.1128/mBio.00388-19 (PMC6495373; doi:10.1128/mBio.00388-19)
Supplement: TABLE S5 [file mBio.00388-19-st005.docx]

**Table S5:**

Genome assembly statistics. N50 denotes the median length of contigs.

| **Assembly** | **N50^1^ (bp)** | **Max scaffold length (bp)** | **Min scaffold length (bp)** | **Coverage (X)** | **Protein coding genes** | **Median gene length (AA)** |
| --- | --- | --- | --- | --- | --- | --- |
| Sefale-s04 | 15623 | 50237 | 265 | 47 | 5170 | 382 |
| EBEgT12 | 15592 | 86735 | 896 | 48 | 2702 | 419 |
| EBEgB8 | 4970 | 30350 | 1000 | 240 | 3420 | 393 |
| TZPlpvs7 | 2842 | 1000 | 14214 | 36 | 5648 | 294 |
| TZPlpvs2-8 | 2655 | 20162 | 1000 | 117 | 4846 | 356 |
| RRPrTmd13 | 3314 | 19301 | 1000 | 205 | 3117 | 327 |
| RRPrNerP7 | 3363 | 13502 | 1000 | 208 | 3062 | 325 |

**Comments**

^1^ N50 denotes the median length of contigs.
